# Quantitative assessment of myelin density using [^11^C]MeDAS PET in patients with multiple sclerosis: a first-in-human study

**DOI:** 10.1007/s00259-022-05770-4

**Published:** 2022-04-02

**Authors:** Chris W. J. van der Weijden, Jan F. Meilof, Anouk van der Hoorn, Junqing Zhu, Chunying Wu, Yanming Wang, Antoon T. M. Willemsen, Rudi A. J. O. Dierckx, Adriaan A. Lammertsma, Erik F. J. de Vries

**Affiliations:** 1 Nuclear Medicine and Molecular Imaging, University of Groningen, University Medical Center Groningen, Hanzeplein 1, Groningen, The Netherlands; 2 Department of Biomedical Sciences of Cells and Systems, University of Groningen, University Medical Center Groningen, Hanzeplein 1, 9713GZ Groningen, The Netherlands; 3 Department of Neurology, Martini Ziekenhuis, Groningen, The Netherlands; 4 Radiology, University of Groningen, University Medical Center Groningen, Hanzeplein 1, 9713GZ Groningen, The Netherlands; 5 Department of Radiology, Case Western Reserve University, Cleveland, OH 44106 USA

**Keywords:** Multiple sclerosis, Quantitative myelin imaging, Positron emission tomography, Arterial input function, Demyelination

## Abstract

**Purpose:**

Multiple sclerosis (MS) is a disease characterized by inflammatory demyelinated lesions. New treatment strategies are being developed to stimulate myelin repair. Quantitative myelin imaging could facilitate these developments. This first-in-man study aimed to evaluate [^11^C]MeDAS as a PET tracer for myelin imaging in humans.

**Methods:**

Six healthy controls and 11 MS patients underwent MRI and dynamic [^11^C]MeDAS PET scanning with arterial sampling. Lesion detection and classification were performed on MRI. [^11^C]MeDAS time-activity curves of brain regions and MS lesions were fitted with various compartment models for the identification of the best model to describe [^11^C]MeDAS kinetics. Several simplified methods were compared to the optimal compartment model.

**Results:**

Visual analysis of the fits of [^11^C]MeDAS time-activity curves showed no preference for irreversible (2T3k) or reversible (2T4k) two-tissue compartment model. Both volume of distribution and binding potential estimates showed a high degree of variability. As this was not the case for 2T3k-derived net influx rate (K_i_), the 2T3k model was selected as the model of choice. Simplified methods, such as SUV and MLAIR2 correlated well with 2T3k-derived K_i_, but SUV showed subject-dependent bias when compared to 2T3k. Both the 2T3k model and the simplified methods were able to differentiate not only between gray and white matter, but also between lesions with different myelin densities.

**Conclusion:**

[^11^C]MeDAS PET can be used for quantification of myelin density in MS patients and is able to distinguish differences in myelin density within MS lesions. The 2T3k model is the optimal compartment model and MLAIR2 is the best simplified method for quantification.

Trial registration.

NL7262. Registered 18 September 2018.

**Supplementary information:**

The online version contains supplementary material available at 10.1007/s00259-022-05770-4.

## Introduction

Multiple sclerosis (MS) is the most common neurodegenerative disease among young adults. MS is characterized by focal inflammation, which results in demyelinated and neurodegenerative plaques, called lesions [1, 2]. The myelin sheaths around neuronal axons have neuroprotective, axonal metabolic, and neurotransmission support functions [3, 4]. Disturbances in myelin integrity therefore decrease neuronal efficacy and make neurons susceptible to degeneration. Restoration of the myelin sheath would restore neuronal function and promote neuronal survival. This hypothesis has prompted the search for remyelination therapies. However, at present, an accurate biomarker to evaluate the efficacy of such remyelination strategies is missing.

In vivo characterization of myelin density using imaging techniques would be an attractive way for monitoring remyelination therapies, as it enables direct insight in their efficacy. Several magnetic resonance imaging (MRI) techniques for myelin imaging have been developed. Unfortunately, a recent review of these methods concluded that current myelin MRI methods do not have the accuracy required for quantitative monitoring of myelin repair [5]. Another technique that could potentially be used for myelin imaging is positron emission tomography (PET) enabling quantification using specific radiopharmaceuticals [6–9].

The disease process in MS involves inflammation resulting in myelin loss and possibly direct neurodegeneration. Some PET tracers for neuroinflammation have been tested in MS [4, 10, 11]. At present, there are no PET tracers that selectively bind to myelin. However, it has been established that several tracers, originally developed for imaging amyloid, also bind to the beta-pleated sheet structure in myelin. This actually explains the relatively high white matter uptake of [^11^C]PiB in healthy volunteers. As there is no clinical overlap between neurodegenerative diseases associated with amyloid deposition and multiple sclerosis, these tracers are of potential interest for myelin imaging. Several amyloid tracers have been used to depict myelin content in preclinical studies. [^11^C]PiB PET was able to detect demyelination in a primate model of MS, although [^11^C]PiB uptake only moderately correlated with myelin histology [12]. Yet, [^11^C]PiB and its derivatives have recently been used in small clinical studies in MS [6, 7, 13].

Although [^11^C]PiB is already available clinically, as it is already used in the diagnosis of Alzheimer’s disease, animal studies have shown that [^11^C]MeDAS might be more accurate for imaging myelin than [^11^C]PiB [6]. Aside from [^11^C]PiB’s affinity for amyloid-beta depositions, [^11^C]PiB is proposed to bind to the beta sheets of the myelin basic protein (MBP). However, [^11^C]MeDAS has actually been shown to bind to intact MBP, which is a key component of intact myelin [14, 15]. Upon myelin damage, MBP loses its compact structure, resulting in the destruction of the binding site for [^11^C]MeDAS. Therefore, a reduction in [^11^C]MeDAS PET signal is indicative for a reduction in myelin density. To date, however, [^11^C]MeDAS PET has not been used in humans yet.

Therefore, the purpose of this study was to (1) assess whether [^11^C]MeDAS PET can detect differences in myelin density in the human brain and to (2) determine the best method for quantitatively analyzing [^11^C]MeDAS PET data.

## Methods

### Autoradiography

Post-mortem brain tissues of a MS patient in 10% formalin obtained from University Hospitals, Cleveland Medical Center, were cryoprotected sequentially in 10%, 20%, and 30% sucrose. Brain tissues were placed in optimal cutting temperature (O.C.T.) embedding medium and frozen at − 20 °C. Next, brain tissues were sectioned at 20 µm in a cryostat and mounted directly onto Superfrost Plus microscope slides (Fisher Scientific, Hampton, USA). These brain sections were incubated in [^11^C]MeDAS (specific activity of 238,650 MBq/µmol, 10% ethanol in saline, 1295 MBq/mL) for 20 min. The slides were then quickly washed with PBS buffer (10 mM, pH 7.0) 3 times. After being dried by air, the slides were put in a cassette and exposed to film for 10 min. In the meantime, standard Luxol Fast Blue (LFB) staining was performed on adjacent brain tissues.

### Subjects

Eleven MS patients, diagnosed according to the revised McDonald criteria [16], and 6 healthy volunteers (HC) were included in this prospective study. The inclusion criteria were at least 18 years old and, in case of MS, a diagnosis of progressive MS. The following exclusion criteria were applied: pregnancy or breastfeeding; a previous adverse reaction to gadolinium; claustrophobia; a diagnosis of cerebrovascular disease; a clinical history of diminished renal or liver function; participation in a trial and use of investigational medication at the time of the study; the presence of magnetizable materials in the body. Written informed consent was obtained from all study participants. The study was approved by the Medical Ethics Review Committee of the University Medical Center Groningen, METc no. 2018/450, Netherlands, Trial register: Trial NL7262.

### Data acquisition

All participants underwent arterial cannulation of either the radial or ulnar artery in the wrist and a 60-min dynamic [^11^C]MeDAS PET acquisition with arterial blood sampling. [^11^C]MeDAS PET scans were acquired on a Siemens Biograph Vision PET/CT scanner, starting with a low-dose CT for attenuation correction, followed by the [^11^C]MeDAS PET scan, which was acquired in list mode. Individual doses of [^11^C]MeDAS were prepared on site according to GMP quality assurance criteria. [^11^C]MeDAS was injected simultaneously with the start of the 60-min PET scan. The total dose in the injection syringe was 416 ± 90 MBq for HC and 498 ± 87 MBq for MS patients. After measuring the amount of tracer that remained in syringe and tubing, the net injected dose was calculated to be 203 ± 46 MBq for HC and 209 ± 35 for MS patients. Continuous arterial blood sampling was performed with an online detection system at a rate of 5 mL/min for 5 min, followed by a rate of 1.66 mL/min for the remainder of the scan. In addition, 5 manual arterial blood samples of 5 mL each were collected at 10, 20, 30, 45, and 60 min for calibrating the whole blood curve and measuring plasma to whole blood ratios. Furthermore, the manual blood samples were used to calculate percentage of intact tracer, which was subsequently fitted with a hill function in order to generate a metabolite-corrected arterial plasma input function.

PET images were corrected for randoms, dead-time, scatter, decay, and attenuation and reconstructed in 26 frames (1 × 10, 10 × 5, 1 × 10, 2 × 30, 3 × 60, 2 × 150, 4 × 300, and 3 × 600 s) with a voxel size of 0.9 × 0.9 × 0.9. MRI scans were performed on the same day as the PET scan. All MRI scans were acquired on the same 3.0 Tesla scanner (Siemens Magnetom Prisma) equipped with a 64-channel head coil. The imaging protocol included the following sequences: a sagittal 3D T1w MPRAGE (TR: 2300 ms; TE: 2.31 ms; TI: 900 ms; flip angle: 8°; slice thickness: 0.9 mm; voxel size: 0.9 × 0.9 × 0.9 mm), a sagittal 3D T2w-FLAIR (TR: 5000 ms; TE: 392 ms; TI: 1800 ms; flip angle: 90°, slice thickness: 0.9 mm; voxel size: 0.9 × 0.9 × 0.9 mm), a sagittal 3D T2w (TR: 3200 ms; TE: 408 ms; flip angle: 90°; slice thickness: 0.9 mm; voxel size: 0.4 × 0.4 × 0.9 mm), and a post-gadolinium sagittal 3D T1w MPRAGE with parameters identical to those of pre-contrast 3D T1w.

### Data analysis

**Fig. 1:**
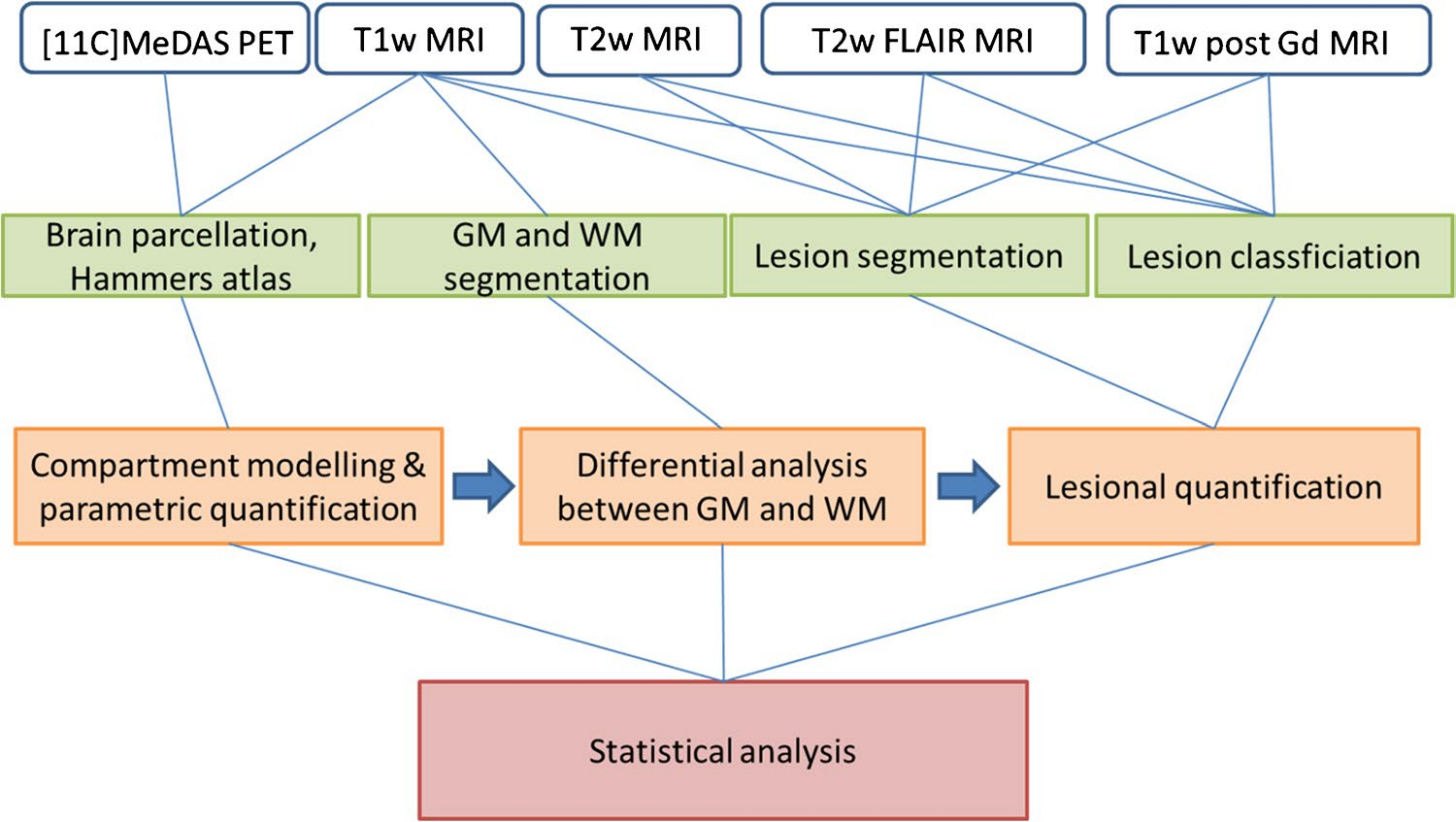
Overview of the data processing

**Fig. 2:**
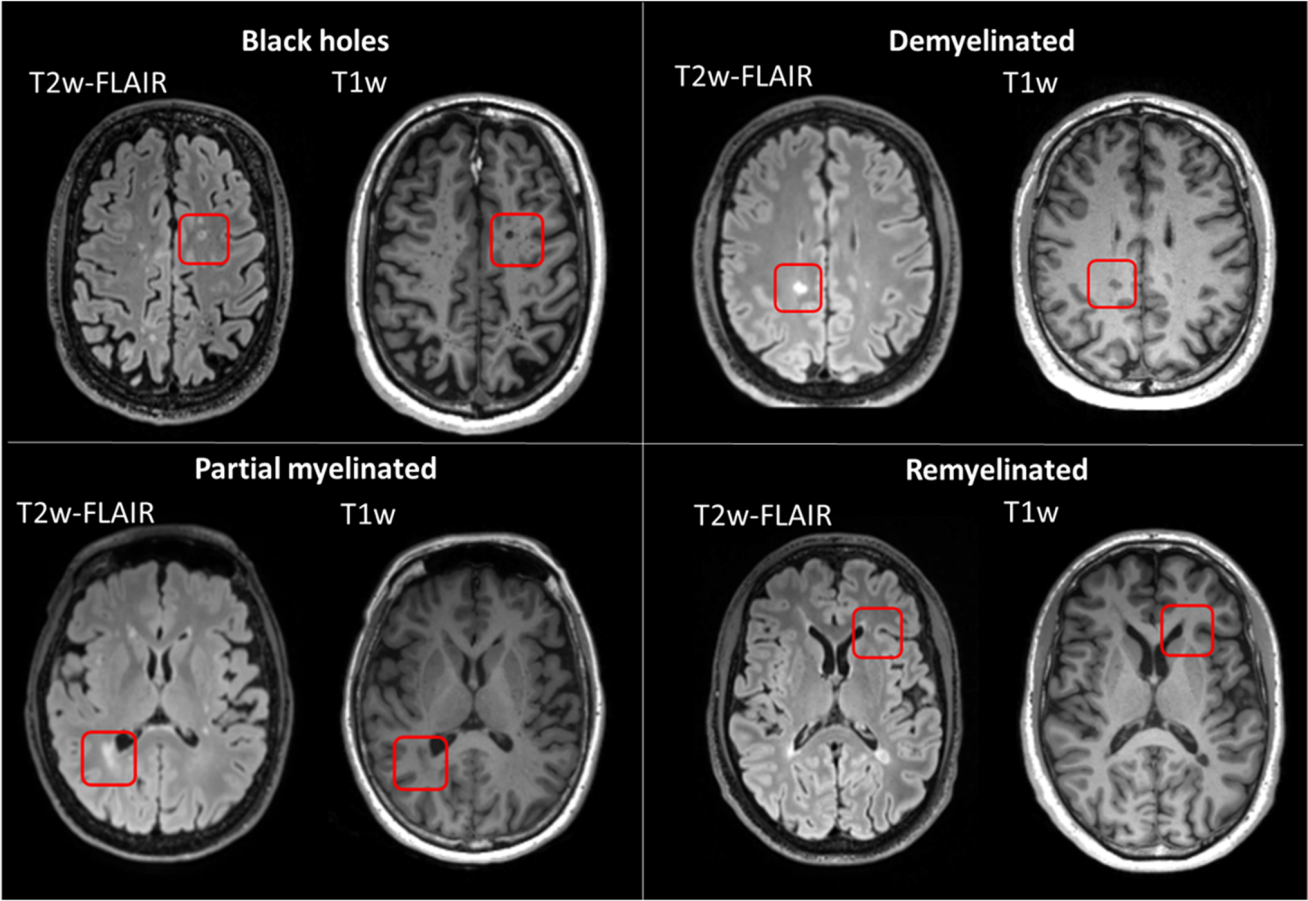
Lesion classification system based on myelin density

Lesions (Fig. 1) were radiologically identified and classified using T1w, T2w, T2w-FLAIR, and T1w post-contrast MRI. When lesions were hyperintense on T1w post-contrast MRI, they were classified as active lesions [17]. Furthermore, lesions were characterized based on their expected myelin density (Fig. 2), as validated using histopathological data by Barkhof et al. [18, 19], and for black holes based in the study of Sahraian et al. [18]. T2w and T2w-FLAIR hyperintense lesions that were iso-intense to normal-appearing white matter (NAWM) on T1w were classified as remyelinated lesions; hypointense T1w lesions that had a smaller lesion volume on T1w compared with T2w and T2w-FLAIR were classified as partial myelinated lesions; hypointense T1w lesions of the same volume as on T2w and T2w-FLAIR images were classified as fully demyelinated lesions; and iso-intense T1w lesions compared with CSF were called black holes and classified as demyelinated and neurodegenerative lesions. Only lesions with at least one diameter larger than 3 mm were considered in this study.

Tissue segmentation was performed to derive binary masks representing white matter lesions (WML), peri-lesions, contra-lateral NAWM, white matter (WM), and gray matter (GM). PMOD (v4.1, PMOD Technologies, Zurich, Switzerland) was used for manual segmentation of WML, peri-lesions, and contra-lateral NAWM. Manual segmentation of WML was performed using the T2w-FLAIR MRI scan. Subsequently, contralateral NAWM regions were generated by assessing the absence of any structural damage using T1w and T2w-FLAIR. Peri-lesions were generated by applying lesion growing of 3 voxels and subsequently subtracting the WML. GM and WM volumes of interest (VOIs) were generated by performing T1w segmentation using SPM12 with a threshold of ≥ 0.9 [20]. A total NAWM volume of interest (VOI) was acquired by extracting the segmented lesion VOIs from the WM VOI.

Within the PMOD software package (v4.1), [^11^C]MeDAS PET images were corrected for movement and co-registered to the T1-weighted MRI using rigid matching. Movement of < 5 mm was considered to be minimal for which no motion correction was performed, but movement between > 5 mm in any direction was subjected to motion correction. Scan motion was determined to be < 10 mm for all subjects. Subsequently, time-activity curves (TACs) in anatomically delineated VOIs based on the Hammers brain atlas [21] were generated. These VOIs were aggregated into frontal lobe, temporal lobe, occipital lobe, parietal lobe, and basal ganglia according to their anatomical location. These VOIs, together with the cerebellum, corpus callosum, thalamus, and brainstem derived from the Hammers atlas, were subdivided between gray matter (GM) and white matter (WM), and between left and right, where applicable. Using these VOI TACs (total 26) and the whole blood and metabolite-corrected plasma curves, [^11^C]MeDAS kinetic analysis was performed, investigating the reversible 1-tissue compartment model (1T2k) and the irreversible (2T3k) and reversible (2T4k) 2-tissue compartment models, all including a parameter for fractional blood volume (V_B_).

Subsequently, the efficacy of several graphical methods (Patlak graphical analysis, MLAIR1 and MLAIR2) was investigated by comparing the net influx rate (K_i_) estimates with K_i_ estimates derived from the 2T3k model using the same VOIs for all methods. Patlak graphical analysis is susceptible to bias due to noisy data and requires the determination of the time interval until equilibrium is reached to select the data points for linear regression. For MLAIR, on the other hand, all data points are used for K_i_ estimations [22]. Consequently, MLAIR is less prone to bias than Patlak analysis. Nonetheless, MLAIR1 remains somewhat susceptible to noise and therefore performs better on a VOI level than on a voxel level. Because MLAIR2 directly calculates K_i_ from the multiple linear regression, these estimates should be stable and robust even at a voxel level. A more detailed description and the exact formulas of Patlak graphical analysis, MLAIR1, and MLAIR2 can be found in the article by Kim and colleagues [22]. In addition, standardized uptake values (SUV) were calculated for the intervals 40–50 min and 50–60 min by correcting tissue uptake for net injected dose and body weight. Next, these SUV were compared with K_i_ to assess whether SUV, which can be derived from a static scan, would be accurate enough in lesion differentiation to substitute kinetic analyses. Finally, [^11^C]MeDAS PET lesion quantification was performed using both compartment models and simplified methods.

### Statistical analysis

The Akaike information criterion (AIC) was used to determine the optimal compartment model for describing [^11^C]MeDAS kinetics across the VOIs derived from the Hammers atlas and subsequently for WML. The model with the highest frequency of lowest AIC per VOI was selected as the preferred model. The correspondence of kinetic parameters with graphical methods and SUV was investigated using Pearson correlation analysis. Next, both kinetic parameters and SUV were assessed on their ability to differentiate between GM and WM using parametric tests, where applicable. Subsequently, the ability of the various parameters to differentiate between lesions that were radiologically characterized into different myelin densities was compared with NAWM using the Mann–Whitney *U* test. Compartment modeling produced parameter estimates with a percent standard error (SE) for each individual brain region in each subject (PMOD v4.1). BP_ND_, V_T_, and K_i_ estimates for a particular brain region in an individual subject with %SE exceeding an arbitrary threshold of 25% were considered unreliable and omitted from further analysis.

Prior to analysis, normality was assessed according to Kolmogorov–Smirnov, using a *p* < 0.05 for non-normal distributed data. When applicable, equal variance was assumed according to Levene’s test for equality of variance, using a *p* < 0.05 for lack of equal variances. All statistical analyses were performed in SPSS and differences were considered to be significant at *p* < 0.05.

## Results

### *Autoradiographic characterization of [*^*11*^*C]MeDAS binding to post-mortem tissue*

**Fig. 3:**
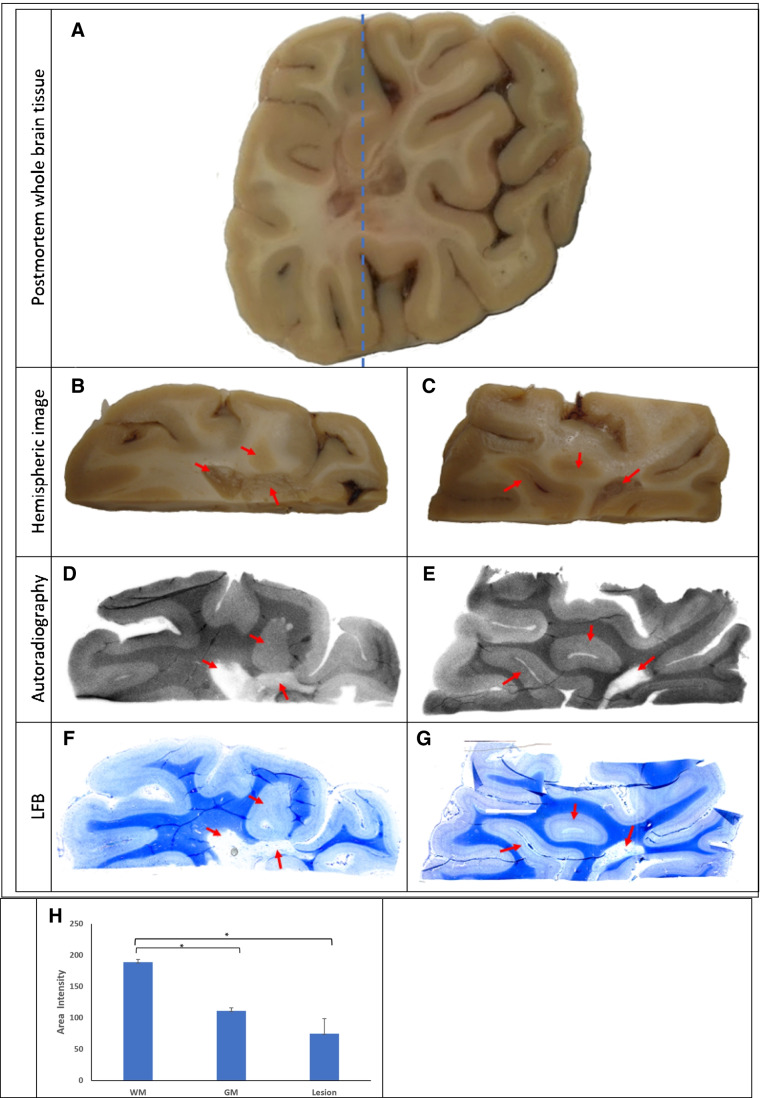
Comparative distribution of [^11^C]MeDAS binding sites on whole brain post-mortem tissue containing demyelinated lesions in white matter: **A** image of a post-mortem whole brain slice showing lesions in white matter; **B**–**C** images of two separate hemispheric regions with several white matter lesions (arrows); **D**–**E** corresponding autoradiograms showing a significant decrease of [^11^C]MeDAS uptake in the lesions; **F**–**G** subsequent LFB staining of adjacent sections confirming demyelination in the lesions; **H** quantitative analysis of the autoradiography of [^11^C]MeDAS on sections of the human brain tissue showing significant reduction of binding in the gray matter and lesion regions compared to the normally myelinated white matter

In preparation for the first-in-human clinical trial, the binding of [^11^C]MeDAS to myelin was characterized by film autoradiography in sections of fresh-frozen post-mortem tissue of a patient with extensive demyelination in the brain (Fig. 3A–C). These autoradiograms showed a heterogeneous distribution of [^11^C]MeDAS uptake, which corresponded with the myelin density in the brain (Fig. 3D–E, H). A high density of binding sites was detected in heavily myelinated white matter regions (189 ± 11; *n* = 6) with much less binding of [^11^C]MeDAS in the less densely myelinated cortical gray matter regions (111 ± 19; *n* = 6) and lesions (75 ± 58; *n* = 6). This distribution pattern of [^11^C]MeDAS was identical to the staining of adjacent sections using Luxol Fast Blue (LFB) (Fig. 3F–G), suggesting that [^11^C]MeDAS binds to myelin in the brain. Consequently, the binding of [^11^C]MeDAS decreased significantly in demyelinated regions, which corresponded with LFB staining results.

### Subjects

Six HC and eleven MS patients were recruited in this study. For one MS patient, radiolabeled metabolites could not be measured due to HPLC malfunction and in another MS patient, the arterial cannula required for blood sampling could not be placed. This resulted in 9 evaluable MS patients, of which 5 had primary and 4 had secondary progressive MS. The 9 MS patients had a mean age of 51.8 (± 8.7), whereas HC had a mean age of 50.6 (± 4.9) and no visible cerebral lesions on MRI. No significant differences were observed between HC and MS with respect to age and sex. Within MS patients, a total of 292 lesions (average 32 ± 28 lesions/patient) were identified, covering a total volume of 63 ml. No lesions were hyperintense on T1w post gadolinium MRI scans, and therefore, all lesions were classified as inactive lesions.

### *[*^*11*^*C]MeDAS distribution in blood and tissue*

**Fig. 4:**
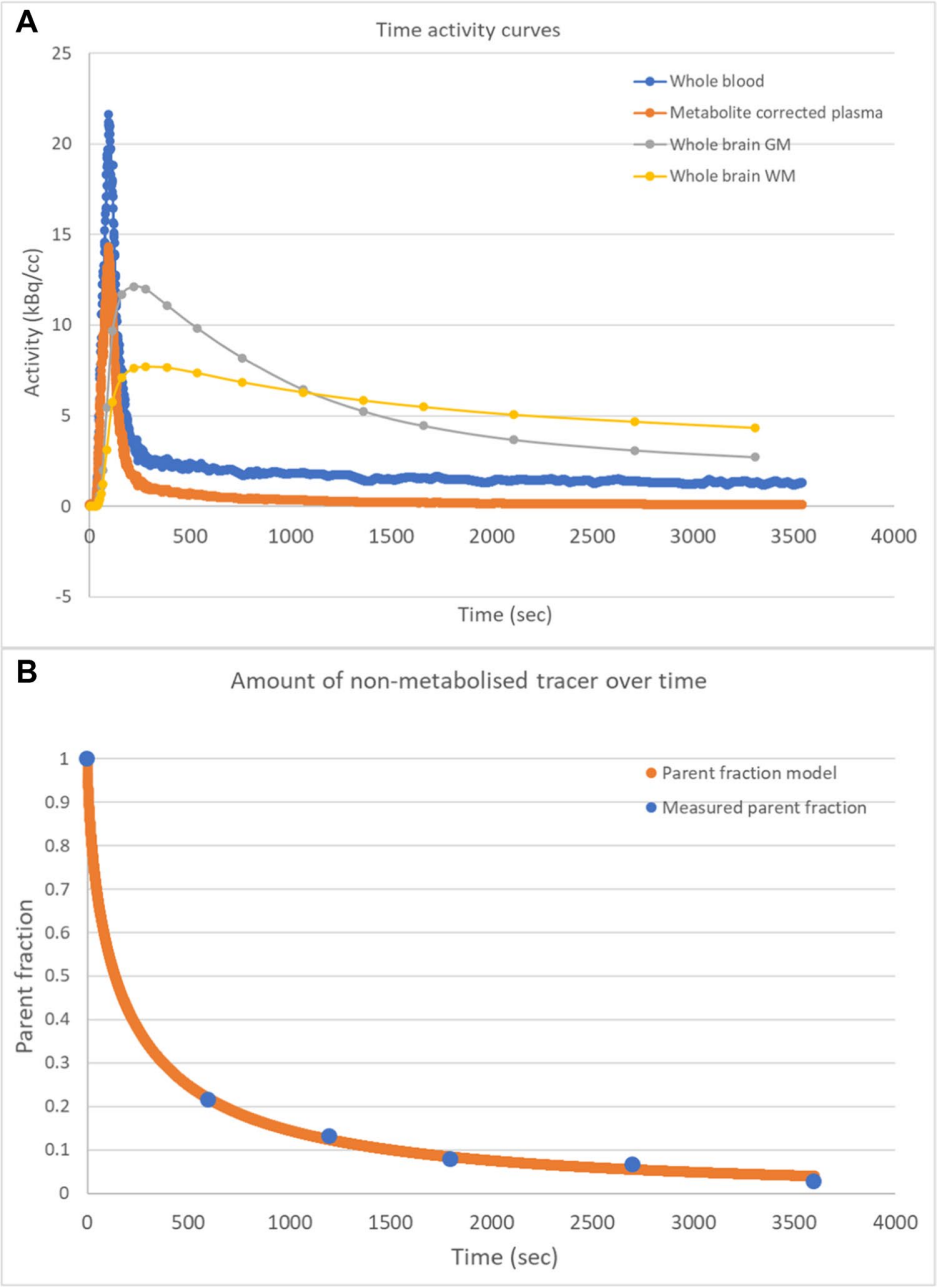
Representative time-activity curves for **A** whole blood, metabolite-corrected plasma, whole brain GM, and whole brain WM and **B** intact tracer in plasma fitted with a hill function

Metabolite analysis (Fig. 4) showed fast metabolism of [^11^C]MeDAS with only 19.4 ± 6.9% of intact tracer left after 10 min, which eventually stabilized around 5%. In addition, high influx of tracer was observed in whole brain GM with a gradual washout (Fig. 4). As expected, for whole brain WM, influx of tracer was less than the GM. More importantly, WM washout was much slower than GM washout, suggestive for myelin-mediated tracer binding in WM. Individual non-metabolized plasma tracer concentration curves are displayed in Supplementary Fig. 1.

### Model comparison

**Fig. 5:**
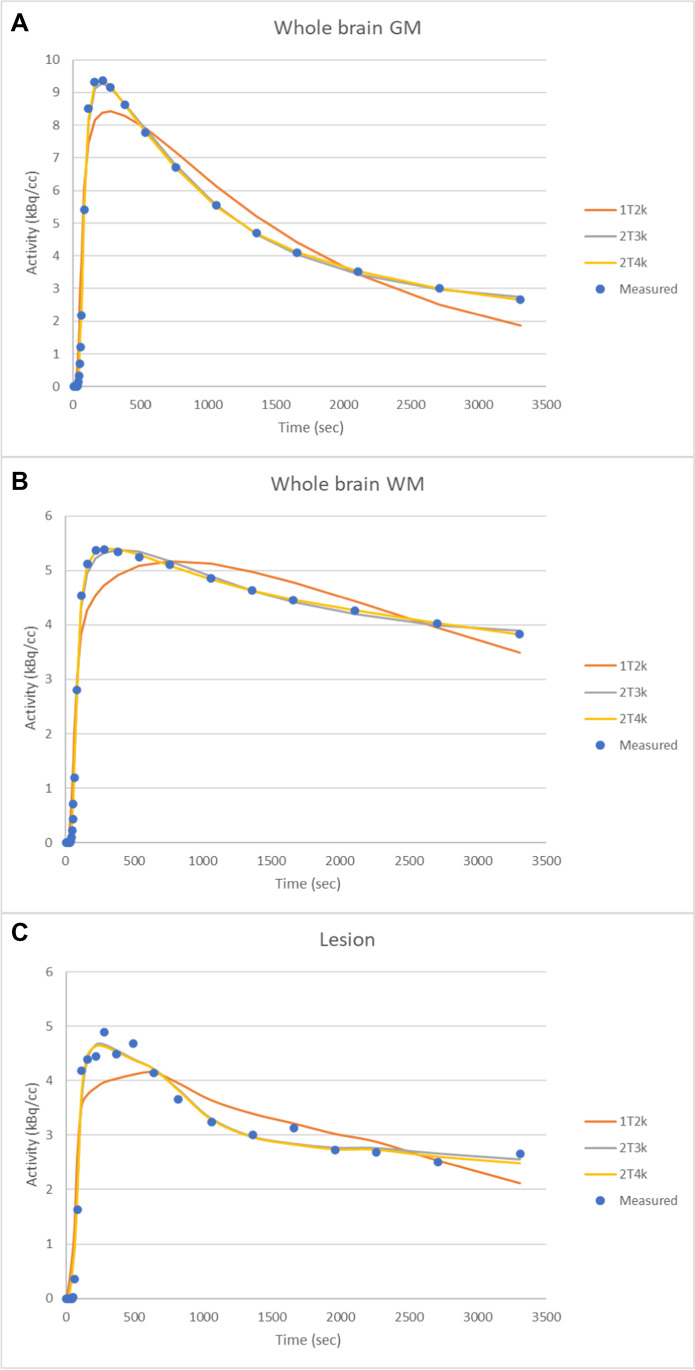
Representative model fits of **A** whole brain GM, **B** whole brain WM, and **C** an MS lesion using 1T2k, 2T3k, and 2T4k models

The fits of the 1T2k, 2T3k, and 2T4k models for whole brain GM, whole brain WM, and a lesion are displayed in Fig. 5. In general, the 1T2k model was not able to describe the kinetic behavior of the tracer, as demonstrated by the sum of squared residuals (Supplementary Table 3) and the goodness of fit (*R*^2^; Supplementary Table 4). In contrast, both 2T3k and 2T4k models performed well. The individual rate constant estimates and standard errors obtained with these models are summarized in Supplementary Tables 5 and 6, respectively.

**Fig. 6:**
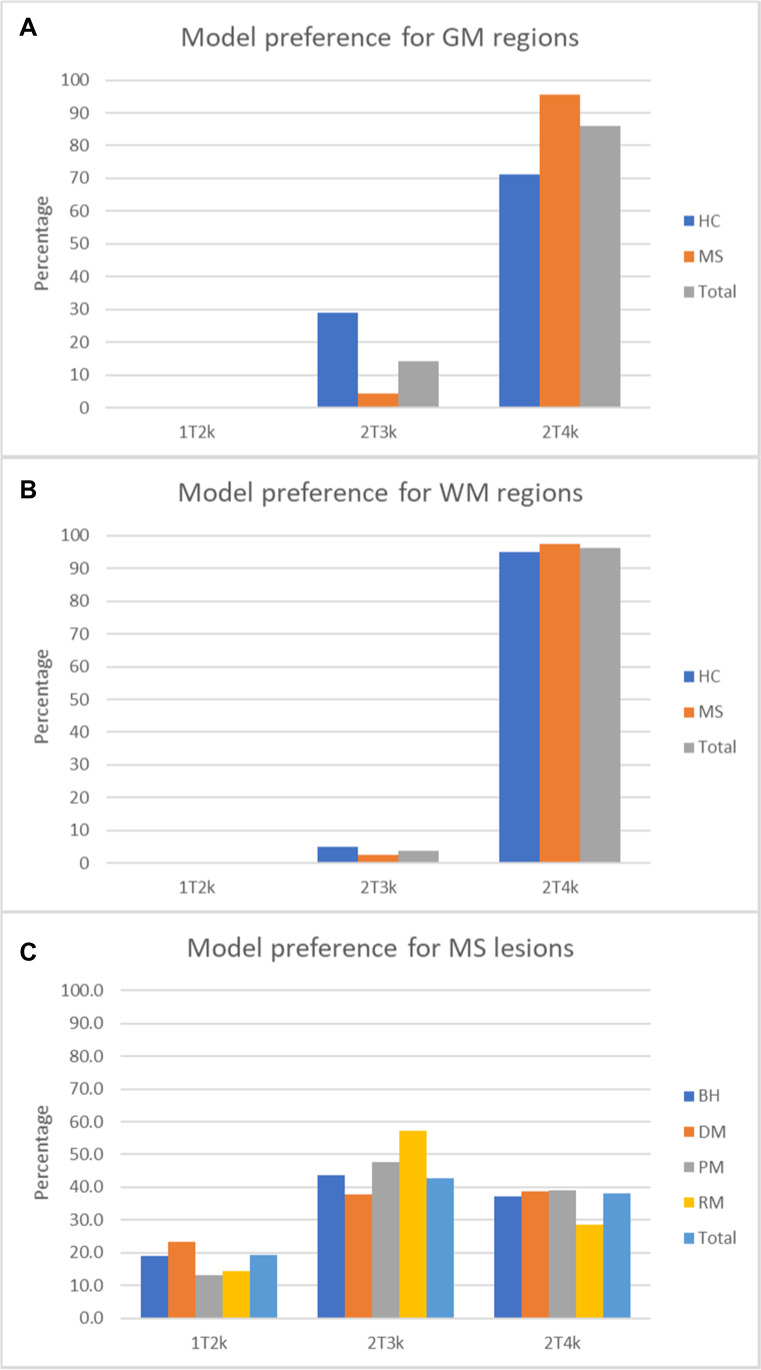
Model preference according to the Akaike information criterion for **A** GM regions, **B** WM regions, and **C** MS lesions. HC, healthy control, MS, multiple sclerosis, GM, gray matter, WM, white matter, BH, black holes, DM, demyelinated lesions, PM, partial myelinated lesions, RM, remyelinated lesions

Using AIC, the frequency of model preference per region derived from the Hammers atlas was calculated, resulting in the 2T4k model as preferred model for [^11^C]MeDAS quantification (Fig. 6). However, 2T4k derived V_T_ and BP_ND_ estimates had a SE > 25% for 11.0% and 15.7% of the regions, respectively. On the other hand, for 1T2k-derived V_T_ and for 2T3k-derived K_i_, there were no regions with an SE > 25%. Interestingly, the difference in AIC values was relatively small between 2T3k and 2T4k models. In contrast to the 2T3k model, the 2T4k model resulted in a large percentage of microparameter estimates with a SE > 25%, in particular k3 and k4 (Supplementary Tables 7, 8, 9, and 10), which may explain the poorer identifiability of 2T4k-derived V_T_ and BP_ND_.

For white matter lesions (WML), the 2T3k model showed the highest preference frequency (Fig. 6). Both 2T3k (29.7% for K_i_) and 2T4k (53.2% for V_T_ and 86.3% for BP_ND_) had a high number of lesions (Table 1) with inaccurate estimates (SE > 25%). Fixing V_B_ at 0.02 (which is the mean V_B_ of white matter [23]) and/or K1/k2 ratio to the whole brain WM value resulted in a reduction in uncertain estimates (i.e., lower SE) for the 2T3k model but did not have much impact on the uncertainty of 2T4k-derived V_T_ and BP_ND_ estimates. Furthermore, 2T3k K_i_ estimates (when fixing either K1/k2 to the whole brain WM value or both K1/k2 and V_B_) showed high correlations (Table 2) with the original (no parameters fixed) 2T3k K_i_ estimates (*r* = 0.95, slope = 1.04; *r* = 0.93, slope = 1.02, respectively). Based on these findings, the 2T3k model with both K1/k2 fixed to the whole brain WM value and either V_B_ fixed to 0.02 or free V_B_ was used for WML quantification.

**Table 1:** Percentage of lesions with unreliable modeling estimates (SE > 25%) with or without fixing V_B_ and/or K_1_/k_2_

	None	V_B_	K_1_/k_2_	K_1_/k_2_ + V_B_
1T2k V_T_	7.8%	6.8%	n.a	n.a
2T3k K_i_	29.7%	27.6%	11.3%	11.9%
2T4k V_T_	53.2%	47.1%	53.6%	51.5%
2T4k BP_ND_	86.3%	81.6%	62.5%	62.1%

*V_*T*_, volume of distribution; *K*_*i*_, net influx rate; *BP*_*ND*_, binding potential; *V*_*B*_, volume of blood fraction.

**Table 2:** Correlation of parameter estimates using models with fixed V_B_ and/or K1/k2, as compared with the original models

	V_B_			K_1_/k_2_			K_1_/k_2_ + V_B_		
	*r*	Slope	Intercept	*r*	Slope	Intercept	*r*	Slope	Intercept
1T2k V_T_	1.00	0.99	0.07	n.a	n.a	n.a	n.a	n.a	n.a
2T3k K_i_	1.00	0.98	0.00	0.95	1.04	-0.01	0.93	1.02	-0.01
2T4k V_T_	1.00	0.96	0.42	0.99	1.00	0.08	0.98	0.98	0.36
2T4k BP_ND_	0.97	0.95	0.07	0.72	0.40	1.23	0.73	0.40	1.29

*V_T_, volume of distribution; *K*_*i*_, net influx rate; *BP*_*ND*_, binding potential; *V*_*B*_, volume of blood fraction.

### Simplified quantification methods

**Fig. 7:**
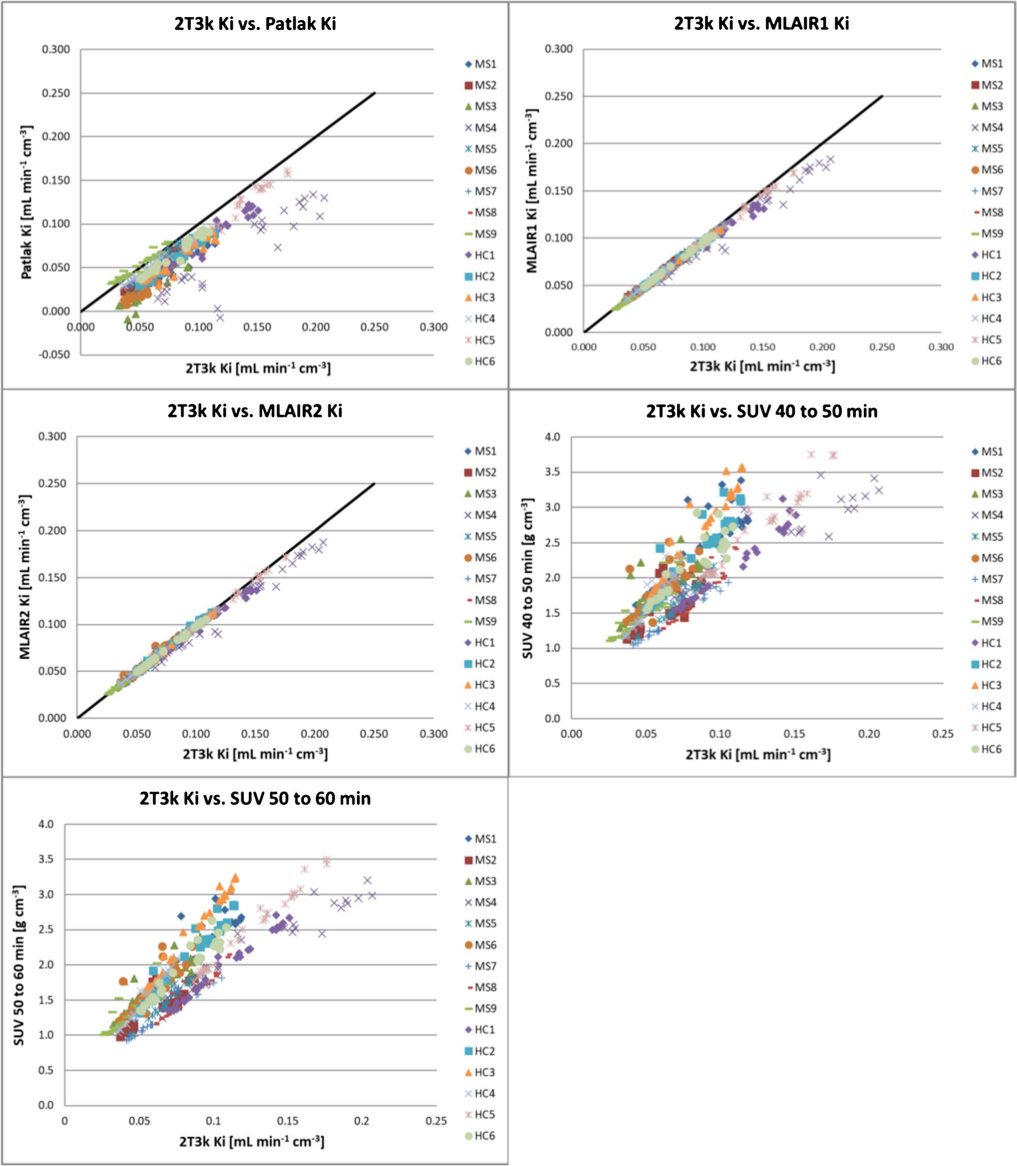
Correlation of K_i_ from graphical methods and SUV with the K_i_ derived from the 2T3k model. The black line is the line of identity. Each data point represents a brain region from an individual subject

The performance of Patlak graphical analysis, MLAIR1, and MLAIR2 was assessed by correlating their K_i_ estimates with those derived from the 2T3k model (Table 3; Fig. 7). All three graphical methods produced K_i_ values that correlated well with 2T3k-derived K_i_ (Patlak *r* = 0.87, MLAIR1 *r* = 0.99, and MLAIR2 *r* = 0.99). In addition, the SUV values from 40 to 50 min and from 50 to 60 min were evaluated (Supplementary Tables 1 and 2). Strong correlations were observed between SUV estimates and 2T3k-derived K_i_ (*r* = 0.83 for SUV from 40 to 50 min and *r* = 0.86 for SUV from 50 to 60 min), but subject-dependent bias when compared to 2T3k K_i_ was observed. Among the simplified methods, only Patlak K_i_ showed a slightly poorer correlation with 2T3k K_i_ in MS patients than in HC. This might be due to [^11^C]MeDAS kinetics being better described by multiple linear regression than a single linear regression, as variations in fitting start time did not improve the accuracy of the Patlak K_i_. A comparison of Patlak analysis with 2T3k and Logan analysis with 2T4k can be found in Supplementary Fig. 2.

**Table 3:** Correlations between K_i_ values derived from simplified methods with those for the original 2T3k model over all brain regions

Simplified method	Controls			MS			All subjects		
	*r*	Slope	Intercept	*r*	Slope	Intercept	*r*	Slope	Intercept
SUV 40–50 min	0.84	n.a	0.87	0.82	n.a	0.85	0.83	n.a	0.81
SUV 50–60 min	0.87	n.a	0.70	0.85	n.a	0.74	0.86	n.a	0.69
Patlak Ki	0.97	0.93	− 0.01	0.80	0.63	0.00	0.87	0.78	0.00
MLAIR1 Ki	1.00	0.94	0.00	0.99	0.89	0.00	0.99	0.91	0.00
MLAIR2 Ki	1.00	0.95	0.00	0.99	0.90	0.01	0.99	0.93	0.00

*K_*i*_, net influx rate; *n.a*., not applicable.

**Fig. 8:**
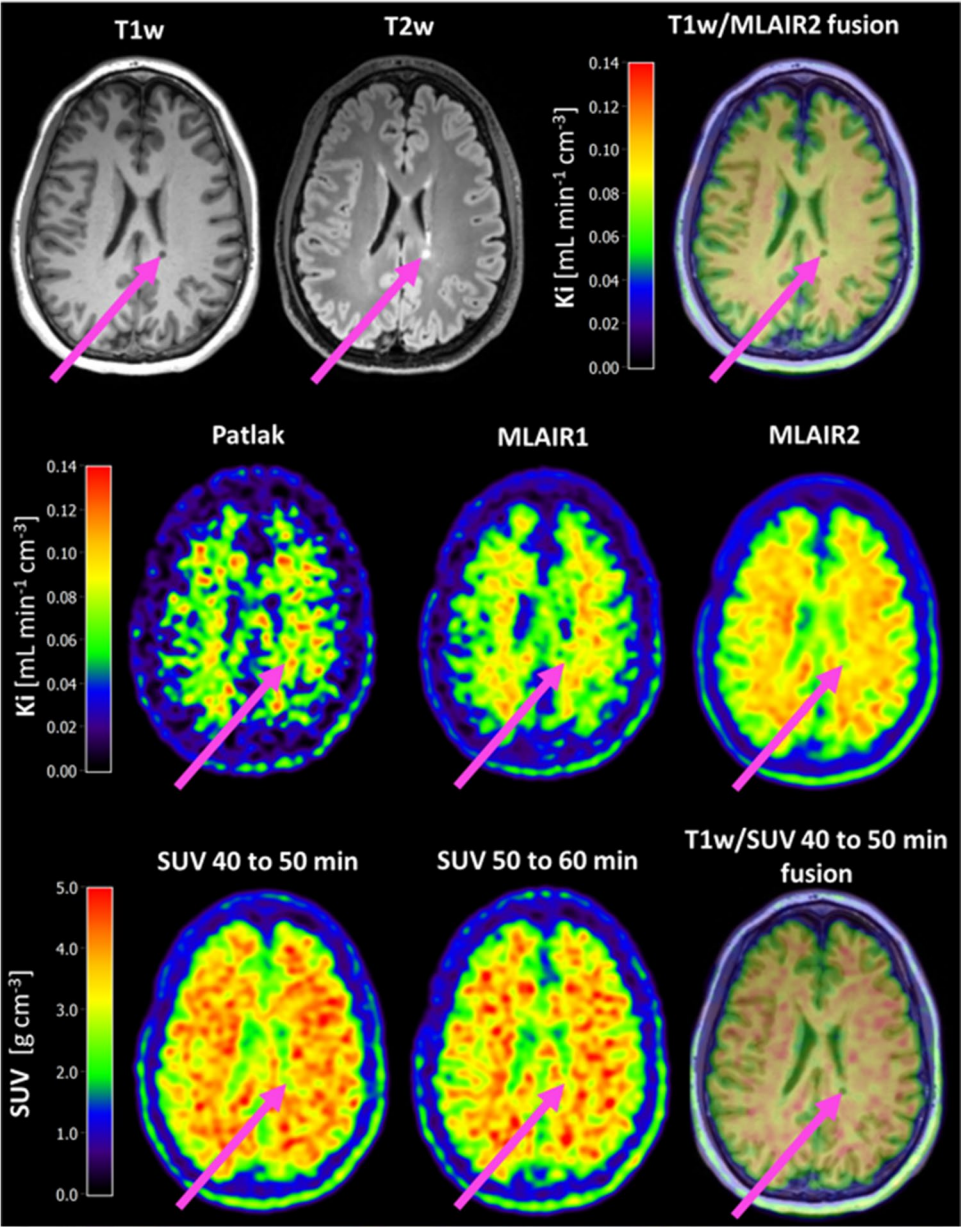
[^11^C]MeDAS PET images. The pink arrow indicates a MS lesion, which was hypo-intense on T1w MRI and hyper-intense on T2w-MRI. Decreased [^11^C]MeDAS uptake can be observed in MLAIR2, SUV 40–50 min, and SUV 50–60 min images, whereas the K_i_ images of Patlak and MLAIR1 are too noisy to clearly differentiate differences in myelin density. For the presentation purpose, the PET images were smoothed with a 4-mm Gaussian filter.

Additional qualitative assessment of the simplified methods showed that both Patlak and MLAIR1 produced very noisy images (Fig. 8). Therefore, only SUV 40–50 min, SUV 50–60 min, and MLAIR2 were considered for further assessment.

### Differential analysis between GM and WM

To determine which quantitative analysis methods and estimated parameters can distinguish between whole brain GM and whole brain WM, quantitative measurements illustrative for tracer binding were assessed in HC. K_i_ derived from the 2T3k model and MLAIR2 and SUV of 40–50 min and SUV of 50–60 min were all able to differentiate between GM and WM (Table 4).

**Table 4:** Differential analysis between whole brain GM and whole brain WM using HC

	GM mean (± SD)	WM mean (± SD)	*T*	Degrees of freedom	*p* value
2T3k K_i_ [mL min^−1^ cm^−3^]	0.064 ± 0.018	0.113 ± 0.031	− 3.4	10	0.007
MLAIR2 K_i_ [mL min^−1^ cm^−3^]	0.062 ± 0.018	0.111 ± 0.029	− 3.5	10	0.005
SUV of 40–50 min [g cm^−3^]	1.71 ± 0.26	2.74 ± 0.44	− 4.9	10	0.001
SUV of 50–60 min [g cm^−3^]	1.53 ± 0.24	2.56 ± 0.42	− 5.1	10	< 0.001

### Lesion analysis

When lesions were classified based on myelin density (Fig. 2; Table 5), 105 lesions were identified as black holes (BH), 111 as demyelinated lesions (DM), 69 as partial myelinated lesions (PM), and 7 as remyelinated lesions (RM). Quantitative PET outcome parameters were assessed for their ability to differentiate lesions from NAWM. Outcome parameters from the 2T3k models were estimated, using K_1_/k_2_ fixed to the K_1_/k_2_ value of whole brain WM and either free V_B_ or V_B_ fixed to 0.02. In addition, MLAIR2, SUV 40–50 min, and SUV 50–60 min were also investigated. When comparing parameters in lesions with those in contra-lateral NAWM (Table 6), all models showed significant differences for black holes, demyelinated lesions, and partial myelinated lesions, but not for remyelinated lesions. The absence of significant differences between remyelinated lesions and NAWM might be because myelin density is largely restored in these lesions.

**Table 5:** Lesion characteristics separated on myelin density

	Black holes	Demyelinated lesions	Partial myelinated lesions	Remyelinated lesions
Total number (*n*)	105	111	69	7
Total volume (mL)	18.1	37.5	7.0	0.28
Average volume (mL)	0.17 ± 0.27	0.34 ± 1.26	0.10 ± 0.13	0.040 ± 0.023

**Table 6:** Comparison of outcome parameters in WML versus contra-lateral NAWM for different analysis methods

Method	Type of lesion	Lesion	NAWM	*U*	*p* value
2T3k + fixed K_1_/k_2_					
K_i_ [mL min^−1^ cm^−3^]	BH	0.072 ± 0.035	0.094 ± 0.039	6544	< 0.001
	DM	0.079 ± 0.038	0.108 ± 0.049	7785	< 0.001
	PM	0.085 ± 0.042	0.106 ± 0.041	3075	< 0.001
	RM	0.102 ± 0.054	0.118 ± 0.050	25	0.63
2T3k + fixed K_1_/k_2_ and V_B_					
K_i_ [mL min^−1^ cm^−3^]	BH	0.071 ± 0.036	0.094 ± 0.039	6601	< 0.001
	DM	0.079 ± 0.039	0.109 ± 0.049	7773	< 0.001
	PM	0.089 ± 0.043	0.107 ± 0.041	3115	< 0.001
	RM	0.097 ± 0.053	0.118 ± 0.051	26	0.53
MLAIR2					
K_i_ [mL min^−1^ cm^−3^]	BH	0.079 ± 0.034	0.102 ± 0.042	7837	< 0.001
	DM	0.089 ± 0.048	0.113 ± 0.052	8368	< 0.001
	PM	0.093 ± 0.045	0.115 ± 0.054	3128	< 0.001
	RM	0.106 ± 0.059	0.135 ± 0.066	33	0.32
SUV of 40–50 min [g cm^−3^]					
	BH	1.65 ± 0.53	2.04 ± 0.52	8055	< 0.001
	DM	1.74 ± 0.53	2.24 ± 0.60	9125	< 0.001
	PM	1.82 ± 0.56	2.19 ± 0.57	3332	< 0.001
	RM	1.91 ± 0.64	2.31 ± 0.67	35	0.21
SUV of 50–60 min [g cm^−3^]					
	BH	1.58 ± 0.44	1.97 ± 0.49	8048	< 0.001
	DM	1.65 ± 0.51	2.13 ± 0.66	8940	< 0.001
	PM	1.77 ± 0.54	2.10 ± 0.45	3299	< 0.001
	RM	1.58 (± 0.68)	1.92 (± 0.52)	32	0.38

*2T3k + fixed K_1_/k_2_, the 2T3k model with a K_1_/k_2_ fixed to the whole brain WM K_1_/k_2_ value, 2T3k + fixed K_1_/k_2_ and V_B_, the 2T3k model with both K_1_/k_2_ and V_B_ fixed to the whole brain WM K_1_/k_2_ and V_B_ value, *U*, the test statistic of the Mann–Whitney *U* test.

## Discussion

Accurate measurements of myelin density would aid in treatment monitoring and facilitate early diagnosis and assessment of prognosis of patients with MS. Therefore, this study aimed to investigate whether [^11^C]MeDAS PET could be used to measure myelin density and, if so, to determine the optimal quantification method. The preclinical autoradiographic characterization in post-mortem brain tissue showed that [^11^C]MeDAS binding corresponded with myelin density. The subsequent PET study showed that [^11^C]MeDAS data were best analyzed using a 2T3k model, and that fixation of the K_1_/k_2_ ratio seemed to produce robust estimates of myelin density. Furthermore, the simplified method MLAIR2 and SUV estimates correlated well with results of the 2T3k model, with MLAIR2 having the highest correlation and was also qualitatively accurate. As compared with NAWM, decreased tracer uptake was observed across lesions, except in remyelinated lesions.

Unfortunately, no patients with active lesions were included, which most likely is a result of enrolling only patients with progressive MS. The inclusion of RRMS would probably have resulted in the inclusion of more patients with active lesions, as the prevalence of active lesions is higher in RRMS than in progressive MS [24]. The inclusion of RRMS patients would probably also have resulted in a larger sample of remyelinated lesions, as this is more prevalent in RRMS. For this proof-of-concept study, however, only patients with progressive MS were enrolled, as demyelination is more prevalent in this group. In addition, the presence of active inflammatory lesions with a disturbed blood brain barrier could have introduced unwanted tracer uptake or clearance.

From animal studies, the optimal compartment model was defined as the 2T4k model [6]. Therefore, a 2T4k model was also expected to be the preferred model in humans. However, PET tracer kinetics may vary across species, and therefore, the findings regarding tracer kinetics in animals are not always directly translatable to humans. Visual inspection of the [^11^C]MeDAS PET model fits showed only minor differences between the 2T3k and 2T4k models. When assessing the optimal compartment model according to AIC, the 2T4k model seemed to be the preferred model, but it provided a substantially higher number of ill-determined parameter estimates than the 1T2k and 2T3k models. Within lesions, the precision of 2T4k V_T_ and BP_ND_ estimates was also relatively low and could not be improved adequately by fixing K_1_/k_2_ and/or V_B_. Logan graphical analysis gave similar results as the 2T4k model (Supplementary Fig. 2) but did not improve the precision of V_T_ estimates. In contrast, the precision of 2T3k derived K_i_ estimates improved significantly with K_1_/k_2_ fixation. The inadequacy of the 2T4k model to quantify myelin density within lesions could be due to the scan duration. It seems plausible that a duration of 60 min is too short for accurate estimation of the k_4_, and therefore, a longer scan might be needed. To support this hypothesis, PET data were re-analyzed for shorter acquisition times (40 and 50 min). A reduced accuracy of the 2T4k parameters V_T_ and BP_ND_ was observed for shorter scanning times (Supplementary Table 11). On the other hand, when acquisition times longer than 60 min are used, radioactivity levels may become so low that proper signal-to-noise ratios and reliable metabolite analyses are no longer guaranteed due to the short half-life of carbon-11, resulting in a reduced accuracy of parameter estimates. In contrast, shortening the acquisition time did not substantially affect the precision of the K_i_ estimates obtained from the 2T3k model (Supplementary Table 12). Nevertheless, shortening the acquisition time tended to slightly increase K_i_ estimates across all brain regions (Supplementary Table 13), but K_i_ values obtained from 60-min acquisition data were still highly correlated with those obtained from 50 min (*r* 0.87; *p* < 0.001; slope = 1.01; intercept = 0.013) and 40 min (*r* 0.87; *p* < 0.001; slope = 1.07; intercept = 0.015) acquisition data. So, the present results indicate that, despite the introduction of some bias, it is possible to quantify myelin density using K_i_ using relatively short acquisition times. Longer scans would only increase patient burden and may not necessarily improve reliability of parameter estimates, mainly because obtaining reliable labeled metabolite estimates at later time points is difficult. Moreover, the 2T3k model with fixation of K_1_/k_2_ was able to differentiate between lesions and NAWM, except for remyelinated lesions. This seems logical, as remyelinated lesions should have myelin densities close to the values of NAWM. Thus, the 2T3k model was considered to be the most appropriate model, as it produced more robust estimates than the 2T4k model. As the lesions were rather small (0.22 ± 0.80 mL), they most likely suffer from partial volume effects. Therefore, a correction of partial volume effects may lead to higher contrast between lesions and NAWM.

The simplified methods MLAIR2 and SUV provided estimates that correlated well with the 2T3k estimates and produced qualitatively good images, but the subject-dependent bias of the SUV when compared to 2T3k Ki indicates that SUV might be less suitable for inter-subject comparisons. Therefore, MLAIR2 could be used as an alternative for accurate and precise quantification, necessary for therapeutic drug development, whereas SUV could possibly be used for lesion identification. However, to substantiate the use of SUV for lesion identification, simulation studies have to be performed to assess the effect of confounders on SUV, as SUV estimates differ upon alterations of cardiovascular throughput, tracer clearance, and tracer excretion. Irrespective of those findings, MLAIR2 has the highest correlation with 2T3k model, is devoid of potential confounders regarding cardiovascular throughput, tracer clearance, and tracer excretion, and could therefore be used as an alternative quantification method for [^11^C]MeDAS PET.

Current clinical research on myelin imaging with PET uses repurposed amyloid-beta PET tracers, such as [^11^C]PiB and [^18^F]florbetaben, in combination with a reference tissue model for quantification [13, 25–29]. One of these studies found a decreased tracer uptake in NAWM of MS patients as compared to HC WM [27], whereas two other studies did not [13, 26]. With [^11^C]MeDAS PET, we also did not find a decreased tracer uptake in NAWM of MS patients as compared to HC WM (data not shown). This would suggest that the myelin outside of MS lesions is preserved from MS pathology, or at least not severely enough affected to be detected. Furthermore, the PET studies with an amyloid tracer showed a decrease in the tracer uptake in MS lesions as compared with NAWM, which illustrates the potential of amyloid PET tracers for myelin imaging [13, 25–29]. [^11^C]MeDAS PET seems to perform comparably with the amyloid PET tracers for assessing the total lesion burden. Although in previous PET studies with amyloid tracers decreases in tracer uptake in WML have been observed [13, 25, 27–29], no differentiation was made between lesion types with different myelin densities. In the present study, a reduced tracer uptake was observed in all types of lesions, except in remyelinated lesions. In addition, there was a relationship between [^11^C]MeDAS uptake and degree of (expected) myelin density within lesions (BH < DM < PM < RM lesions; Table 6). The observed differences in tracer binding between lesions with different levels of myelin content support the specificity of [^11^C]MeDAS as a myelin tracer. It also supports the potential use of [^11^C]MeDAS binding as an accurate in vivo quantitative measure of myelin content. Furthermore, a longitudinal pilot study with [^11^C]PiB already illustrated the potential of PET imaging for detecting temporal changes in myelin content [13], while this still remains to be investigated for [^11^C]MeDAS PET. More detailed comparisons between tracers are difficult, because the published studies have used different patient populations of a highly heterogenous disease and a different quantification method. While we used compartment modeling, the amyloid tracer studies did not acquire blood data and used therefore reference tissue methods. However, the use of a reference tissue method requires the absence of target expression in the reference region and stability of the K_1_/k_2_ across the whole brain. As MS lesions, in particular active lesions, can have alterations in the blood–brain barrier integrity, it is likely that reference tissue methods may be less suitable for analyzing MS data and therefore have not been investigated in this study. A prospective study directly comparing an amyloid tracer and [^11^C]MeDAS in the same patients with the same scanning protocol and analysis method would enable a more thorough comparison of the tracers. Until such studies have been performed, no firm conclusion can be drawn regarding which tracer would be most specific and accurate.

In conclusion, [^11^C]MeDAS PET can be used to differentiate between myelin densities of GM and WM and also between lesions that are radiologically categorized according to myelin density. The 2T3k model with fixed K_1_/k_2_ seems to be the preferred method for quantification, with K_i_ being the most accurate parameter for estimating myelin density. Furthermore, MLAIR2 seems to be both qualitatively and quantitatively accurate and precise enough to be an alternative for the 2T3k model for [^11^C]MeDAS PET quantification. Due to [^11^C]MeDAS ability to estimate differences in myelin densities across lesions with different myelin densities, [^11^C]MeDAS PET might be a potential quantitative biomarker for efficacy evaluation of remyelination therapies.

## Supplementary information

Below is the link to the electronic supplementary material.

Supplementary file1 (DOCX 331 KB)

## Abbreviations

1T2k: Reversible 1 tissue compartment model
2T3k: Irreversible 2 tissue compartment model
2T4k: Reversible 2 tissue compartment model
AIC: Akaike information criterion
BH: Black hole
BP_ND_: Binding potential
DM: Demyelinated lesion
GM: Gray matter
HC: Healthy controls
K_i_: Net influx rate
LFB: Luxol Fast Blue
MLAIR: Multiple linear analysis for irreversible radiotracers
MRI: Magnetic resonance imaging
MS: Multiple sclerosis
NAWM: Normal appearing white matter
PET: Positron emission tomography
PM: Partial demyelinated/remyelinated lesion
RM: Remyelinated lesion
SUV: Standardized uptake value
V_B_: Blood volume fraction
V_T_: Volume of distribution
WM: White matter
WML: White matter lesion
