# Supplementary material for: Quantitative assessment of myelin density using [11C]MeDAS PET in patients with multiple sclerosis: a first-in-human study
Source: Eur J Nucl Med Mol Imaging. 2022 Apr 2;49(10):3492–507. doi: 10.1007/s00259-022-05770-4 (PMC9308583; doi:10.1007/s00259-022-05770-4)
Supplement: Supplementary file 1 — Supplementary file1 (DOCX 331 KB) [file 259_2022_5770_MOESM1_ESM.docx]

Supplementary Table 1: Mean and standard deviation of the SUV per brain region at 50 to 60 minutes after tracer injection

| **Region** | **SUV** [g cm^-3^] | | | **Standard deviation** | | |
| --- | --- | --- | --- | --- | --- | --- |
|  | **HC** | **MS** | **Total** | **HC** | **MS** | **Total** |
| Whole_brain_GM | 1.49 | 1.26 | 1.35 | 0.24 | 0.21 | 0.24 |
| Thalamus_l | 2.25 | 1.87 | 2.02 | 0.39 | 0.35 | 0.40 |
| Thalamus_r | 2.04 | 1.76 | 1.87 | 0.32 | 0.49 | 0.44 |
| Cerebellum_l | 1.38 | 1.16 | 1.25 | 0.25 | 0.20 | 0.24 |
| Cerebellum_r | 1.41 | 1.11 | 1.23 | 0.26 | 0.14 | 0.24 |
| Frontal_lobe_GM_L | 1.56 | 1.32 | 1.41 | 0.24 | 0.22 | 0.25 |
| Temporal_lobe_GM_L | 1.44 | 1.21 | 1.30 | 0.27 | 0.21 | 0.25 |
| Parietal_lobe_GM_L | 1.62 | 1.35 | 1.46 | 0.21 | 0.23 | 0.25 |
| Occipital_lobe_GM_L | 1.55 | 1.34 | 1.42 | 0.22 | 0.20 | 0.23 |
| Frontal_lobe_GM_R | 1.59 | 1.33 | 1.43 | 0.26 | 0.23 | 0.27 |
| Temporal_lobe_GM_R | 1.43 | 1.20 | 1.29 | 0.25 | 0.21 | 0.25 |
| Parietal_lobe_GM_R | 1.59 | 1.32 | 1.43 | 0.22 | 0.23 | 0.25 |
| Occipital_lobe_GM_R | 1.53 | 1.32 | 1.41 | 0.24 | 0.20 | 0.24 |
| Basal_ganglia_L | 1.86 | 1.56 | 1.68 | 0.33 | 0.26 | 0.32 |
| Basal_ganglia_R | 1.85 | 1.47 | 1.62 | 0.31 | 0.29 | 0.34 |
| Whole_brain_WM | 2.49 | 1.97 | 2.18 | 0.41 | 0.44 | 0.49 |
| Corp_Callosum | 2.29 | 1.64 | 1.90 | 0.48 | 0.37 | 0.52 |
| Brainstem | 2.70 | 2.13 | 2.36 | 0.46 | 0.49 | 0.55 |
| Cerebellum_l_wm | 2.74 | 2.20 | 2.42 | 0.48 | 0.48 | 0.54 |
| Cerebellum_r_wm | 2.70 | 2.12 | 2.35 | 0.53 | 0.42 | 0.54 |
| Frontal_lobe_WM_L | 2.50 | 1.98 | 2.19 | 0.43 | 0.46 | 0.51 |
| Temporal_lobe_WM_L | 2.25 | 1.70 | 1.92 | 0.38 | 0.35 | 0.45 |
| Parietal_lobe_WM_L | 2.47 | 1.92 | 2.14 | 0.42 | 0.45 | 0.51 |
| Occipital_lobe_WM_L | 2.22 | 1.82 | 1.98 | 0.33 | 0.36 | 0.39 |
| Frontal_lobe_WM_R | 2.52 | 1.98 | 2.19 | 0.44 | 0.48 | 0.52 |
| Temporal_lobe_WM_R | 2.21 | 1.71 | 1.91 | 0.38 | 0.40 | 0.46 |
| Parietal_lobe_WM_R | 2.46 | 1.89 | 2.12 | 0.38 | 0.45 | 0.50 |
| Occipital_lobe_WM_R | 2.21 | 1.81 | 1.97 | 0.30 | 0.39 | 0.40 |

*GM = grey matter, WM = white matter, R = right, L = left

Supplementary Table 2: Mean and standard deviation of the SUV per brain region at 40 to 50 minutes after tracer injection.

| **Region** | **SUV** [g cm^-3^] | | | **SUV SD** | | |
| --- | --- | --- | --- | --- | --- | --- |
|  | **HC** | **MS** | **Total** | **HC** | **MS** | **Total** |
| Whole_brain_GM | 1.67 | 1.40 | 1.51 | 0.26 | 0.24 | 0.27 |
| Thalamus_l | 2.67 | 2.18 | 2.38 | 0.45 | 0.47 | 0.51 |
| Thalamus_r | 2.42 | 2.02 | 2.18 | 0.32 | 0.59 | 0.53 |
| Cerebellum_l | 1.57 | 1.30 | 1.41 | 0.27 | 0.23 | 0.27 |
| Cerebellum_r | 1.59 | 1.26 | 1.39 | 0.29 | 0.18 | 0.27 |
| Frontal_lobe_GM_L | 1.72 | 1.44 | 1.55 | 0.25 | 0.26 | 0.29 |
| Temporal_lobe_GM_L | 1.61 | 1.35 | 1.46 | 0.28 | 0.25 | 0.28 |
| Parietal_lobe_GM_L | 1.77 | 1.48 | 1.59 | 0.23 | 0.26 | 0.29 |
| Occipital_lobe_GM_L | 1.71 | 1.46 | 1.56 | 0.22 | 0.23 | 0.25 |
| Frontal_lobe_GM_R | 1.75 | 1.45 | 1.57 | 0.28 | 0.27 | 0.30 |
| Temporal_lobe_GM_R | 1.61 | 1.34 | 1.45 | 0.28 | 0.23 | 0.28 |
| Parietal_lobe_GM_R | 1.77 | 1.45 | 1.58 | 0.21 | 0.26 | 0.29 |
| Occipital_lobe_GM_R | 1.68 | 1.46 | 1.55 | 0.24 | 0.23 | 0.25 |
| Basal_ganglia_L | 2.08 | 1.74 | 1.88 | 0.34 | 0.33 | 0.36 |
| Basal_ganglia_R | 2.09 | 1.68 | 1.84 | 0.34 | 0.34 | 0.39 |
| Whole_brain_WM | 2.68 | 2.11 | 2.34 | 0.43 | 0.49 | 0.53 |
| Corp_Callosum | 2.38 | 1.72 | 1.98 | 0.48 | 0.39 | 0.53 |
| Brainstem | 3.07 | 2.41 | 2.67 | 0.49 | 0.56 | 0.61 |
| Cerebellum_l_wm | 2.99 | 2.44 | 2.66 | 0.53 | 0.54 | 0.59 |
| Cerebellum_r_wm | 2.97 | 2.33 | 2.59 | 0.53 | 0.45 | 0.57 |
| Frontal_lobe_WM_L | 2.68 | 2.09 | 2.33 | 0.45 | 0.51 | 0.56 |
| Temporal_lobe_WM_L | 2.40 | 1.84 | 2.07 | 0.40 | 0.41 | 0.48 |
| Parietal_lobe_WM_L | 2.62 | 2.03 | 2.26 | 0.42 | 0.48 | 0.53 |
| Occipital_lobe_WM_L | 2.38 | 1.93 | 2.11 | 0.37 | 0.39 | 0.43 |
| Frontal_lobe_WM_R | 2.68 | 2.09 | 2.33 | 0.45 | 0.53 | 0.57 |
| Temporal_lobe_WM_R | 2.37 | 1.82 | 2.04 | 0.37 | 0.41 | 0.47 |
| Parietal_lobe_WM_R | 2.62 | 1.99 | 2.24 | 0.42 | 0.48 | 0.55 |
| Occipital_lobe_WM_R | 2.38 | 1.92 | 2.10 | 0.32 | 0.40 | 0.43 |

*GM = grey matter, WM = white matter, R = right, L = left

Supplementary Table 3: The mean (standard deviation) of the sum of squared residuals (also known as sum of squared unweighted residuals) per compartment model per brain region

| **Region** | **Sum of squared residuals** | | |
| --- | --- | --- | --- |
|  | **1T2k** | **2T3k** | **2T4k** |
| Whole_brain_GM | 97.3 (75.3) | 17.9 (15.3) | 14.9 (15.1) |
| Thalamus_l | 82.8 (65.0) | 23.8 (18.1) | 17.2 (16.8) |
| Thalamus_r | 84.6 (65.2) | 25.0 (18.3) | 18.4 (17.0) |
| Cerebellum_l | 68.8 (62.6) | 15.7 (13.8) | 12.7 (13.3) |
| Cerebellum_r | 67.3 (62.2) | 15.1 (13.4) | 12.3 (12.8) |
| Frontal_lobe_GM_L | 125.0 (107.1) | 20.7 (18.2) | 17.2 (17.4) |
| Temporal_lobe_GM_L | 65.5 (57.1) | 14.1 (12.3) | 11.7 (12.0) |
| Parietal_lobe_GM_L | 111.4 (91.1) | 18.6 (16.6) | 15.7 (16.1) |
| Occipital_lobe_GM_L | 97.8 (78.7) | 17.5 (16.0) | 15.2 (16.0) |
| Frontal_lobe_GM_R | 127.8 (111.1) | 20.8 (18.5) | 17.1 (17.6) |
| Temporal_lobe_GM_R | 65.7 (57.8) | 13.8 (12.1) | 11.5 (11.9) |
| Parietal_lobe_GM_R | 110.5 (93.8) | 18.4 (16.8) | 15.7 (16.4) |
| Occipital_lobe_GM_R | 99.3 (79.2) | 17.5 (16.0) | 15.2 (16.1) |
| Basal_ganglia_L | 96.7 (81.7) | 19.3 (17.1) | 15.6 (16.4) |
| Basal_ganglia_R | 92.6 (79.4) | 20.5 (18.1) | 15.8 (16.3) |
| Whole_brain_WM | 30.7 (25.8) | 6.9 (5.8) | 4.5 (4.7) |
| Corp_Callosum | 12.3 (11.1) | 3.3 (2.9) | 1.9 (2.1) |
| Brainstem | 40.7 (36.0) | 12.7 (10.3) | 8.8 (8.8) |
| Cerebellum_l_wm | 50.0 (40.3) | 13.5 (10.8) | 8.9 (8.9) |
| Cerebellum_r_wm | 48.5 (39.8) | 13.1 (10.7) | 8.7 (8.5) |
| Frontal_lobe_WM_L | 32.4 (27.6) | 6.6 (5.4) | 4.2 (4.3) |
| Temporal_lobe_WM_L | 28.3 (25.3) | 6.0 (5.1) | 4.3 (4.5) |
| Parietal_lobe_WM_L | 34.9 (30.4) | 6.3 (5.4) | 4.3 (4.4) |
| Occipital_lobe_WM_L | 55.4 (48.5) | 9.2 (8.3) | 7.3 (7.7) |
| Frontal_lobe_WM_R | 30.8 (23.7) | 6.6 (5.4) | 4.0 (4.1) |
| Temporal_lobe_WM_R | 29.0 (24.9) | 6.1 (5.3) | 4.5 (4.7) |
| Parietal_lobe_WM_R | 33.8 (26.5) | 6.2 (5.1) | 4.3 (4.2) |
| Occipital_lobe_WM_R | 55.4 (49.3) | 9.2 (8.3) | 7.3 (7.9) |
|  |  |  |  |
| Total | 67.0 (68.6) | 13.7 (13.8) | 10.7 (12.8) |

*GM = grey matter, WM = white matter, R = right, L = left

Supplementary Table 4: The mean (standard deviation) of the R^2^ (also known as goodness of fit) per compartment model per brain region

| **Region** | **R^2^ (goodness of fit)** | | |
| --- | --- | --- | --- |
|  | **1T2k** | **2T3k** | **2T4k** |
| Whole_brain_GM | 0.87 (0.07) | 0.97 (0.03) | 0.98 (0.03) |
| Thalamus_l | 0.92 (0.05) | 0.97 (0.03) | 0.98 (0.02) |
| Thalamus_r | 0.92 (0.05) | 0.97 (0.03) | 0.98 (0.02) |
| Cerebellum_l | 0.89 (0.07) | 0.97 (0.03) | 0.98 (0.03) |
| Cerebellum_r | 0.89 (0.07) | 0.97 (0.03) | 0.98 (0.03) |
| Frontal_lobe_GM_L | 0.85 (0.09) | 0.97 (0.03) | 0.98 (0.03) |
| Temporal_lobe_GM_L | 0.90 (0.06) | 0.97 (0.03) | 0.98 (0.03) |
| Parietal_lobe_GM_L | 0.86 (0.08) | 0.97 (0.03) | 0.98 (0.03) |
| Occipital_lobe_GM_L | 0.87 (0.07) | 0.97 (0.03) | 0.98 (0.03) |
| Frontal_lobe_GM_R | 0.85 (0.09) | 0.97 (0.03) | 0.98 (0.03) |
| Temporal_lobe_GM_R | 0.89 (0.06) | 0.97 (0.03) | 0.98 (0.03) |
| Parietal_lobe_GM_R | 0.86 (0.08) | 0.97 (0.03) | 0.98 (0.03) |
| Occipital_lobe_GM_R | 0.87 (0.07) | 0.97 (0.03) | 0.98 (0.03) |
| Basal_ganglia_L | 0.89 (0.07) | 0.97 (0.03) | 0.98 (0.03) |
| Basal_ganglia_R | 0.89 (0.07) | 0.97 (0.03) | 0.98 (0.03) |
| Whole_brain_WM | 0.92 (0.04) | 0.98 (0.02) | 0.99 (0.02) |
| Corp_Callosum | 0.93 (0.05) | 0.98 (0.02) | 0.99 (0.02) |
| Brainstem | 0.94 (0.04) | 0.98 (0.02) | 0.98 (0.02) |
| Cerebellum_l_wm | 0.91 (0.04) | 0.97 (0.02) | 0.98 (0.02) |
| Cerebellum_r_wm | 0.91 (0.04) | 0.97 (0.03) | 0.98 (0.02) |
| Frontal_lobe_WM_L | 0.91 (0.04) | 0.98 (0.02) | 0.99 (0.02) |
| Temporal_lobe_WM_L | 0.92 (0.04) | 0.98 (0.02) | 0.99 (0.02) |
| Parietal_lobe_WM_L | 0.90 (0.05) | 0.98 (0.02) | 0.99 (0.02) |
| Occipital_lobe_WM_L | 0.89 (0.05) | 0.98 (0.02) | 0.98 (0.02) |
| Frontal_lobe_WM_R | 0.91 (0.04) | 0.98 (0.02) | 0.99 (0.02) |
| Temporal_lobe_WM_R | 0.91 (0.04) | 0.98 (0.02) | 0.99 (0.02) |
| Parietal_lobe_WM_R | 0.90 (0.05) | 0.98 (0.02) | 0.99 (0.02) |
| Occipital_lobe_WM_R | 0.89 (0.05) | 0.98 (0.02) | 0.98 (0.02) |
|  |  |  |  |
| Total | 0.90 (0.06) | 0.98 (0.03) | 0.98 (0.02) |

*GM = grey matter, WM = white matter, R = right, L = left

Supplementary Table 5: Mean (standard deviation) and mean %SE (standard deviation) of the microparameters estimated with the 2T3k model

| **Region** | **2T3k** | | | | | |
| --- | --- | --- | --- | --- | --- | --- |
|  | **K1** [mL min^-1^ cm^-3^] | | **k2** [min^-1^] | | **k3** [min^-1^] | |
|  | **Mean (SD)** | **Mean %SE (SD)** | **Mean (SD)** | **Mean %SE (SD)** | **Mean (SD)** | **Mean %SE (SD)** |
| Whole_brain_GM | 0.710 (0.267) | 2.07 (0.96) | 0.121 (0.019) | 3.19 (1.57) | 0.011 (0.003) | 6.17 (3.34) |
| Thalamus_l | 0.757 (0.289) | 2.72 (0.89) | 0.077 (0.013) | 4.63 (1.79) | 0.009 (0.003) | 12.86 (8.25) |
| Thalamus_r | 0.787 (0.326) | 2.63 (1.04) | 0.082 (0.014) | 4.38 (2.35) | 0.008 (0.002) | 12.47 (7.74) |
| Cerebellum_l | 0.637 (0.231) | 2.27 (0.99) | 0.112 (0.021) | 3.98 (2.28) | 0.010 (0.003) | 9.09 (5.97) |
| Cerebellum_r | 0.614 (0.207) | 2.26 (0.97) | 0.111 (0.021) | 4.02 (2.25) | 0.010 (0.003) | 9.05 (6.09) |
| Frontal_lobe_GM_L | 0.782 (0.297) | 2.35 (1.17) | 0.134 (0.022) | 3.80 (2.40) | 0.012 (0.003) | 7.64 (5.76) |
| Temporal_lobe_GM_L | 0.630 (0.243) | 2.02 (0.93) | 0.108 (0.019) | 3.61 (2.14) | 0.010 (0.003) | 8.25 (5.95) |
| Parietal_lobe_GM_L | 0.753 (0.289) | 2.23 (1.08) | 0.128 (0.020) | 3.84 (2.27) | 0.012 (0.003) | 7.35 (5.33) |
| Occipital_lobe_GM_L | 0.722 (0.261) | 2.18 (1.12) | 0.123 (0.020) | 3.68 (2.33) | 0.012 (0.003) | 7.31 (5.55) |
| Frontal_lobe_GM_R | 0.782 (0.317) | 2.40 (1.16) | 0.135 (0.022) | 3.93 (2.40) | 0.012 (0.003) | 7.64 (5.88) |
| Temporal_lobe_GM_R | 0.622 (0.237) | 2.01 (0.96) | 0.108 (0.019) | 3.58 (2.21) | 0.010 (0.003) | 8.21 (6.12) |
| Parietal_lobe_GM_R | 0.749 (0.312) | 2.20 (1.07) | 0.130 (0.023) | 3.76 (2.29) | 0.012 (0.003) | 7.32 (5.60) |
| Occipital_lobe_GM_R | 0.727 (0.266) | 2.16 (1.12) | 0.126 (0.021) | 3.57 (2.35) | 0.012 (0.003) | 7.19 (5.63) |
| Basal_ganglia_L | 0.747 (0.303) | 2.35 (1.06) | 0.108 (0.020) | 3.95 (2.22) | 0.012 (0.003) | 8.03 (5.87) |
| Basal_ganglia_R | 0.740 (0.293) | 2.53 (1.12) | 0.107 (0.018) | 4.16 (2.37) | 0.012 (0.003) | 8.39 (5.59) |
| Whole_brain_WM | 0.391 (0.162) | 2.28 (0.80) | 0.063 (0.011) | 6.42 (2.55) | 0.023 (0.004) | 7.98 (3.81) |
| Corp_Callosum | 0.235 (0.099) | 2.72 (0.77) | 0.048 (0.013) | 10.31 (3.37) | 0.033 (0.011) | 11.26 (5.02) |
| Brainstem | 0.507 (0.192) | 2.21 (0.75) | 0.054 (0.011) | 5.45 (2.46) | 0.013 (0.003) | 11.15 (5.82) |
| Cerebellum_l_wm | 0.508 (0.193) | 2.71 (0.68) | 0.064 (0.011) | 6.50 (2.41) | 0.018 (0.004) | 9.60 (3.92) |
| Cerebellum_r_wm | 0.498 (0.187) | 2.57 (0.81) | 0.064 (0.012) | 6.38 (2.50) | 0.018 (0.004) | 9.69 (4.39) |
| Frontal_lobe_WM_L | 0.385 (0.165) | 2.47 (0.93) | 0.067 (0.013) | 7.30 (2.88) | 0.027 (0.005) | 8.10 (3.95) |
| Temporal_lobe_WM_L | 0.378 (0.157) | 2.08 (0.79) | 0.066 (0.011) | 5.63 (2.22) | 0.021 (0.005) | 7.59 (3.68) |
| Parietal_lobe_WM_L | 0.389 (0.163) | 2.35 (0.88) | 0.072 (0.013) | 6.50 (2.67) | 0.027 (0.006) | 7.19 (3.47) |
| Occipital_lobe_WM_L | 0.495 (0.190) | 2.21 (0.89) | 0.088 (0.016) | 4.99 (2.29) | 0.021 (0.004) | 6.35 (3.59) |
| Frontal_lobe_WM_R | 0.381 (0.171) | 2.36 (0.98) | 0.066 (0.013) | 7.00 (2.82) | 0.027 (0.005) | 8.12 (3.61) |
| Temporal_lobe_WM_R | 0.381 (0.159) | 2.02 (0.82) | 0.069 (0.012) | 5.37 (2.48) | 0.021 (0.005) | 7.34 (4.21) |
| Parietal_lobe_WM_R | 0.392 (0.170) | 2.32 (0.85) | 0.073 (0.012) | 6.22 (2.70) | 0.027 (0.005) | 6.78 (3.30) |
| Occipital_lobe_WM_R | 0.492 (0.192) | 2.25 (0.94) | 0.088 (0.016) | 5.10 (2.40) | 0.021 (0.004) | 6.48 (3.75) |

*GM = grey matter, WM = white matter, R = right, L = left

Supplementary Table 6: Mean (standard deviation) and mean %SE (standard deviation) of the microparameters estimated with the 2T4k model

| **Region** | **2T4k** | | | | | | | |
| --- | --- | --- | --- | --- | --- | --- | --- | --- |
|  | **K1** [mL min^-1^ cm^-3^] | | **k2** [min^-1^] | | **k3** [min^-1^] | | **k4** [min^-1^] | |
|  | **Mean (SD)** | **Mean %SE (SD)** | **Mean (SD)** | **Mean %SE (SD)** | **Mean (SD)** | **Mean %SE (SD)** | **Mean (SD)** | **Mean %SE (SD)** |
| Whole_brain_GM | 0.743 (0.267) | 1.89 (1.16) | 0.140 (0.025) | 4.68 (3.43) | 0.022 (0.013) | 14.30 (9.50) | 0.018 (0.012) | 26.21 (21.15) |
| Thalamus_l | 0.814 (0.296) | 2.61 (2.04) | 0.120 (0.064) | 9.19 (8.87) | 0.051 (0.062) | 26.21 (22.29) | 0.040 (0.023) | 24.87 (25.09) |
| Thalamus_r | 0.842 (0.333) | 2.84 (1.80) | 0.118 (0.044) | 9.12 (7.66) | 0.041 (0.038) | 27.49 (21.42) | 0.040 (0.022) | 28.24 (24.67) |
| Cerebellum_l | 0.669 (0.235) | 2.16 (1.24) | 0.133 (0.029) | 6.11 (4.31) | 0.024 (0.015) | 23.38 (21.09) | 0.023 (0.014) | 95.66 (256.44) |
| Cerebellum_r | 0.646 (0.210) | 2.21 (1.26) | 0.133 (0.028) | 6.35 (4.58) | 0.024 (0.014) | 23.40 (20.40) | 0.024 (0.012) | 33.39 (29.07) |
| Frontal_lobe_GM_L | 0.818 (0.300) | 2.11 (1.29) | 0.154 (0.028) | 5.22 (3.58) | 0.022 (0.011) | 17.58 (14.48) | 0.017 (0.012) | 38.62 (38.97) |
| Temporal_lobe_GM_L | 0.656 (0.244) | 1.99 (1.16) | 0.125 (0.024) | 5.53 (4.04) | 0.022 (0.017) | 21.95 (18.64) | 0.020 (0.012) | 40.43 (34.81) |
| Parietal_lobe_GM_L | 0.785 (0.288) | 2.04 (1.27) | 0.145 (0.024) | 5.32 (3.94) | 0.022 (0.011) | 17.67 (15.18) | 0.015 (0.011) | 46.66 (52.07) |
| Occipital_lobe_GM_L | 0.750 (0.259) | 2.15 (1.42) | 0.139 (0.021) | 5.42 (4.15) | 0.021 (0.010) | 18.39 (15.45) | 0.016 (0.009) | 37.98 (31.99) |
| Frontal_lobe_GM_R | 0.819 (0.322) | 2.17 (1.34) | 0.155 (0.029) | 5.26 (4.07) | 0.023 (0.012) | 17.81 (15.89) | 0.018 (0.012) | 39.68 (44.15) |
| Temporal_lobe_GM_R | 0.648 (0.237) | 1.97 (1.21) | 0.126 (0.023) | 5.55 (4.19) | 0.023 (0.017) | 21.11 (18.48) | 0.020 (0.014) | 40.83 (43.27) |
| Parietal_lobe_GM_R | 0.781 (0.317) | 2.17 (1.25) | 0.147 (0.026) | 5.50 (4.27) | 0.022 (0.011) | 17.87 (15.39) | 0.017 (0.012) | 39.44 (40.39) |
| Occipital_lobe_GM_R | 0.755 (0.264) | 2.04 (1.31) | 0.141 (0.021) | 5.21 (4.07) | 0.021 (0.010) | 18.27 (15.77) | 0.016 (0.011) | 44.34 (44.38) |
| Basal_ganglia_L | 0.784 (0.304) | 2.17 (1.25) | 0.129 (0.030) | 5.92 (4.10) | 0.026 (0.015) | 18.76 (16.47) | 0.021 (0.014) | 38.58 (44.66) |
| Basal_ganglia_R | 0.782 (0.297) | 2.13 (1.24) | 0.133 (0.034) | 6.12 (4.19) | 0.028 (0.016) | 18.79 (16.31) | 0.023 (0.012) | 29.98 (38.17) |
| Whole_brain_WM | 0.425 (0.166) | 2.11 (1.41) | 0.099 (0.029) | 8.70 (6.54) | 0.061 (0.024) | 13.90 (11.90) | 0.021 (0.006) | 15.29 (13.71) |
| Corp_Callosum | 0.267 (0.103) | 3.37 (1.97) | 0.106 (0.045) | 15.52 (10.33) | 0.105 (0.036) | 16.64 (13.20) | 0.021 (0.008) | 7.28E+102 (2.82E+103) |
| Brainstem | 0.548 (0.201) | 2.18 (1.19) | 0.091 (0.048) | 10.95 (7.35) | 0.059 (0.054) | 26.59 (20.05) | 0.032 (0.013) | 144.73 (488.23) |
| Cerebellum_l_wm | 0.555 (0.201) | 2.64 (1.38) | 0.104 (0.030) | 11.18 (6.65) | 0.061 (0.024) | 19.10 (11.95) | 0.028 (0.007) | 16.46 (10.48) |
| Cerebellum_r_wm | 0.543 (0.189) | 2.62 (1.56) | 0.105 (0.035) | 11.09 (7.67) | 0.060 (0.028) | 18.82 (13.64) | 0.027 (0.008) | 17.62 (14.00) |
| Frontal_lobe_WM_L | 0.420 (0.168) | 2.24 (1.62) | 0.107 (0.031) | 8.33 (6.80) | 0.065 (0.022) | 12.50 (11.25) | 0.018 (0.006) | 15.43 (13.87) |
| Temporal_lobe_WM_L | 0.404 (0.160) | 2.11 (1.24) | 0.092 (0.025) | 8.18 (5.42) | 0.048 (0.020) | 17.21 (14.24) | 0.018 (0.009) | 111.74 (353.26) |
| Parietal_lobe_WM_L | 0.420 (0.165) | 2.11 (1.47) | 0.106 (0.028) | 8.03 (6.19) | 0.058 (0.017) | 12.68 (11.12) | 0.017 (0.005) | 17.29 (16.18) |
| Occipital_lobe_WM_L | 0.523 (0.191) | 2.03 (1.29) | 0.112 (0.021) | 6.95 (4.87) | 0.039 (0.012) | 14.01 (11.24) | 0.015 (0.006) | 26.60 (23.46) |
| Frontal_lobe_WM_R | 0.420 (0.178) | 2.37 (1.58) | 0.111 (0.037) | 9.18 (6.55) | 0.069 (0.026) | 12.62 (10.74) | 0.019 (0.006) | 14.57 (13.95) |
| Temporal_lobe_WM_R | 0.406 (0.160) | 2.35 (1.65) | 0.095 (0.028) | 8.97 (6.14) | 0.048 (0.023) | 16.66 (12.70) | 0.019 (0.009) | 23.30 (17.14) |
| Parietal_lobe_WM_R | 0.422 (0.173) | 2.34 (1.47) | 0.105 (0.025) | 8.54 (6.01) | 0.056 (0.015) | 13.15 (10.05) | 0.016 (0.005) | 18.80 (16.44) |
| Occipital_lobe_WM_R | 0.518 (0.193) | 2.09 (1.33) | 0.110 (0.022) | 7.04 (5.01) | 0.039 (0.014) | 14.82 (12.11) | 0.014 (0.006) | 29.79 (28.39) |

*GM = grey matter, WM = white matter, R = right, L = left

Supplementary Table 7: %SE assessment of the K1 per brain region as determined with the 2T3k and the 2T4k models. The percentage of subjects with %SE in the K1 >25% or >50% are presented.

| **Region** | **K1** [mL min^-1^ cm^-3^] | | | |
| --- | --- | --- | --- | --- |
|  | **2T3k** | | **2T4k** | |
|  | **%SE>25%** | **%SE>50%** | **%SE>25%** | **%SE>50%** |
| Whole_brain_GM | 0.0 | 0.0 | 0.0 | 0.0 |
| Thalamus_l | 0.0 | 0.0 | 0.0 | 0.0 |
| Thalamus_r | 0.0 | 0.0 | 0.0 | 0.0 |
| Cerebellum_l | 0.0 | 0.0 | 0.0 | 0.0 |
| Cerebellum_r | 0.0 | 0.0 | 0.0 | 0.0 |
| Frontal_lobe_GM_L | 0.0 | 0.0 | 0.0 | 0.0 |
| Temporal_lobe_GM_L | 0.0 | 0.0 | 0.0 | 0.0 |
| Parietal_lobe_GM_L | 0.0 | 0.0 | 0.0 | 0.0 |
| Occipital_lobe_GM_L | 0.0 | 0.0 | 0.0 | 0.0 |
| Frontal_lobe_GM_R | 0.0 | 0.0 | 0.0 | 0.0 |
| Temporal_lobe_GM_R | 0.0 | 0.0 | 0.0 | 0.0 |
| Parietal_lobe_GM_R | 0.0 | 0.0 | 0.0 | 0.0 |
| Occipital_lobe_GM_R | 0.0 | 0.0 | 0.0 | 0.0 |
| Basal_ganglia_L | 0.0 | 0.0 | 0.0 | 0.0 |
| Basal_ganglia_R | 0.0 | 0.0 | 0.0 | 0.0 |
| Whole_brain_WM | 0.0 | 0.0 | 0.0 | 0.0 |
| Corp_Callosum | 0.0 | 0.0 | 0.0 | 0.0 |
| Brainstem | 0.0 | 0.0 | 0.0 | 0.0 |
| Cerebellum_l_wm | 0.0 | 0.0 | 0.0 | 0.0 |
| Cerebellum_r_wm | 0.0 | 0.0 | 0.0 | 0.0 |
| Frontal_lobe_WM_L | 0.0 | 0.0 | 0.0 | 0.0 |
| Temporal_lobe_WM_L | 0.0 | 0.0 | 0.0 | 0.0 |
| Parietal_lobe_WM_L | 0.0 | 0.0 | 0.0 | 0.0 |
| Occipital_lobe_WM_L | 0.0 | 0.0 | 0.0 | 0.0 |
| Frontal_lobe_WM_R | 0.0 | 0.0 | 0.0 | 0.0 |
| Temporal_lobe_WM_R | 0.0 | 0.0 | 0.0 | 0.0 |
| Parietal_lobe_WM_R | 0.0 | 0.0 | 0.0 | 0.0 |
| Occipital_lobe_WM_R | 0.0 | 0.0 | 0.0 | 0.0 |
|  |  |  |  |  |
| Total | 0.0 | 0.0 | 0.0 | 0.0 |

*GM = grey matter, WM = white matter, R = right, L = left

Supplementary Table 8: %SE assessment of the k2 per brain region as determined with the 2T3k and the 2T4k models. The percentage of subjects with %SE in the k2 >25% or >50% are presented.

| **Region** | **k2** [min^-1^] | | | |
| --- | --- | --- | --- | --- |
|  | **2T3k** | | **2T4k** | |
|  | **%SE>25%** | **%SE>50%** | **%SE>25%** | **%SE>50%** |
| Whole_brain_GM | 0.0 | 0.0 | 0.0 | 0.0 |
| Thalamus_l | 0.0 | 0.0 | 6.7 | 0.0 |
| Thalamus_r | 0.0 | 0.0 | 6.7 | 0.0 |
| Cerebellum_l | 0.0 | 0.0 | 0.0 | 0.0 |
| Cerebellum_r | 0.0 | 0.0 | 0.0 | 0.0 |
| Frontal_lobe_GM_L | 0.0 | 0.0 | 0.0 | 0.0 |
| Temporal_lobe_GM_L | 0.0 | 0.0 | 0.0 | 0.0 |
| Parietal_lobe_GM_L | 0.0 | 0.0 | 0.0 | 0.0 |
| Occipital_lobe_GM_L | 0.0 | 0.0 | 0.0 | 0.0 |
| Frontal_lobe_GM_R | 0.0 | 0.0 | 0.0 | 0.0 |
| Temporal_lobe_GM_R | 0.0 | 0.0 | 0.0 | 0.0 |
| Parietal_lobe_GM_R | 0.0 | 0.0 | 0.0 | 0.0 |
| Occipital_lobe_GM_R | 0.0 | 0.0 | 0.0 | 0.0 |
| Basal_ganglia_L | 0.0 | 0.0 | 0.0 | 0.0 |
| Basal_ganglia_R | 0.0 | 0.0 | 0.0 | 0.0 |
| Whole_brain_WM | 0.0 | 0.0 | 0.0 | 0.0 |
| Corp_Callosum | 0.0 | 0.0 | 13.3 | 0.0 |
| Brainstem | 0.0 | 0.0 | 6.7 | 0.0 |
| Cerebellum_l_wm | 0.0 | 0.0 | 6.7 | 0.0 |
| Cerebellum_r_wm | 0.0 | 0.0 | 13.3 | 0.0 |
| Frontal_lobe_WM_L | 0.0 | 0.0 | 0.0 | 0.0 |
| Temporal_lobe_WM_L | 0.0 | 0.0 | 0.0 | 0.0 |
| Parietal_lobe_WM_L | 0.0 | 0.0 | 0.0 | 0.0 |
| Occipital_lobe_WM_L | 0.0 | 0.0 | 0.0 | 0.0 |
| Frontal_lobe_WM_R | 0.0 | 0.0 | 0.0 | 0.0 |
| Temporal_lobe_WM_R | 0.0 | 0.0 | 0.0 | 0.0 |
| Parietal_lobe_WM_R | 0.0 | 0.0 | 0.0 | 0.0 |
| Occipital_lobe_WM_R | 0.0 | 0.0 | 0.0 | 0.0 |
|  |  |  |  |  |
| Total | 0.0 | 0.0 | 1.9 | 0.0 |

*GM = grey matter, WM = white matter, R = right, L = left

Supplementary Table 9: %SE assessment of the k3 per brain region as determined with the 2T3k and the 2T4k models. The percentage of subjects with %SE in the k3 >25% or >50% are presented.

| **Region** | **k3** [min^-1^] | | | |
| --- | --- | --- | --- | --- |
|  | **2T3k** | | **2T4k** | |
|  | **%SE>25%** | **%SE>50%** | **%SE>25%** | **%SE>50%** |
| Whole_brain_GM | 0.0 | 0.0 | 13.3 | 0.0 |
| Thalamus_l | 13.3 | 0.0 | 33.3 | 20.0 |
| Thalamus_r | 6.7 | 0.0 | 40.0 | 13.3 |
| Cerebellum_l | 6.7 | 0.0 | 26.7 | 13.3 |
| Cerebellum_r | 6.7 | 0.0 | 26.7 | 13.3 |
| Frontal_lobe_GM_L | 0.0 | 0.0 | 26.7 | 6.7 |
| Temporal_lobe_GM_L | 6.7 | 0.0 | 26.7 | 13.3 |
| Parietal_lobe_GM_L | 0.0 | 0.0 | 26.7 | 6.7 |
| Occipital_lobe_GM_L | 0.0 | 0.0 | 20.0 | 6.7 |
| Frontal_lobe_GM_R | 0.0 | 0.0 | 20.0 | 6.7 |
| Temporal_lobe_GM_R | 6.7 | 0.0 | 26.7 | 6.7 |
| Parietal_lobe_GM_R | 0.0 | 0.0 | 20.0 | 6.7 |
| Occipital_lobe_GM_R | 0.0 | 0.0 | 26.7 | 6.7 |
| Basal_ganglia_L | 0.0 | 0.0 | 26.7 | 6.7 |
| Basal_ganglia_R | 0.0 | 0.0 | 20.0 | 6.7 |
| Whole_brain_WM | 0.0 | 0.0 | 20.0 | 0.0 |
| Corp_Callosum | 0.0 | 0.0 | 20.0 | 0.0 |
| Brainstem | 0.0 | 0.0 | 26.7 | 13.3 |
| Cerebellum_l_wm | 0.0 | 0.0 | 20.0 | 0.0 |
| Cerebellum_r_wm | 0.0 | 0.0 | 26.7 | 6.7 |
| Frontal_lobe_WM_L | 0.0 | 0.0 | 20.0 | 0.0 |
| Temporal_lobe_WM_L | 0.0 | 0.0 | 20.0 | 6.7 |
| Parietal_lobe_WM_L | 0.0 | 0.0 | 13.3 | 0.0 |
| Occipital_lobe_WM_L | 0.0 | 0.0 | 13.3 | 0.0 |
| Frontal_lobe_WM_R | 0.0 | 0.0 | 13.3 | 0.0 |
| Temporal_lobe_WM_R | 0.0 | 0.0 | 20.0 | 0.0 |
| Parietal_lobe_WM_R | 0.0 | 0.0 | 13.3 | 0.0 |
| Occipital_lobe_WM_R | 0.0 | 0.0 | 13.3 | 0.0 |
|  |  |  |  |  |
| Total | 1.7 | 0.0 | 22.1 | 5.7 |

*GM = grey matter, WM = white matter, R = right, L = left

Supplementary Table 10: %SE assessment of the k4 per brain region as determined with the 2T4k model. The percentage of subjects with %SE in the k4 >25% or >50% are presented.

| **Region** | **k4** [min^-1^] | |
| --- | --- | --- |
|  | **2T4k** | |
|  | **%SE>25%** | **%SE>50%** |
| Whole_brain_GM | 40.0 | 6.7 |
| Thalamus_l | 20.0 | 13.3 |
| Thalamus_r | 33.3 | 20.0 |
| Cerebellum_l | 46.7 | 20.0 |
| Cerebellum_r | 53.3 | 20.0 |
| Frontal_lobe_GM_L | 46.7 | 13.3 |
| Temporal_lobe_GM_L | 53.3 | 20.0 |
| Parietal_lobe_GM_L | 53.3 | 20.0 |
| Occipital_lobe_GM_L | 53.3 | 20.0 |
| Frontal_lobe_GM_R | 46.7 | 13.3 |
| Temporal_lobe_GM_R | 60.0 | 20.0 |
| Parietal_lobe_GM_R | 60.0 | 13.3 |
| Occipital_lobe_GM_R | 60.0 | 20.0 |
| Basal_ganglia_L | 46.7 | 20.0 |
| Basal_ganglia_R | 26.7 | 13.3 |
| Whole_brain_WM | 20.0 | 6.7 |
| Corp_Callosum | 20.0 | 6.7 |
| Brainstem | 26.7 | 13.3 |
| Cerebellum_l_wm | 20.0 | 0.0 |
| Cerebellum_r_wm | 20.0 | 6.7 |
| Frontal_lobe_WM_L | 13.3 | 6.7 |
| Temporal_lobe_WM_L | 33.3 | 20.0 |
| Parietal_lobe_WM_L | 20.0 | 6.7 |
| Occipital_lobe_WM_L | 33.3 | 26.7 |
| Frontal_lobe_WM_R | 13.3 | 6.7 |
| Temporal_lobe_WM_R | 33.3 | 6.7 |
| Parietal_lobe_WM_R | 13.3 | 6.7 |
| Occipital_lobe_WM_R | 33.3 | 20.0 |
|  |  |  |
| Total | 35.7 | 13.8 |

*GM = grey matter, WM = white matter, R = right, L = left

Supplementary Table 11: Assessment of the effect of acquisition time on the accuracy of the outcome parameters V_T_ and BP_ND_ as estimated from the 2T4k model. The percentage of subjects with %SE in the outcome parameter >25% are presented.

| **Outcome parameter** | **V_T_** [mL cm^-3^] | | | **BP_ND_** | | |
| --- | --- | --- | --- | --- | --- | --- |
| **Acquisition time** | **40 min** | **50 min** | **60 min** | **40 min** | **50 min** | **60 min** |
|  |  |  |  |  |  |  |
| Whole_brain_GM | 33.3 | 33.3 | 6.7 | 40.0 | 46.7 | 6.7 |
| Thalamus_l | 13.3 | 13.3 | 0.0 | 33.3 | 20.0 | 13.3 |
| Thalamus_r | 13.3 | 0.0 | 0.0 | 33.3 | 20.0 | 20.0 |
| Cerebellum_l | 20.0 | 13.3 | 20.0 | 33.3 | 40.0 | 20.0 |
| Cerebellum_r | 20.0 | 20.0 | 13.3 | 26.7 | 40.0 | 13.3 |
| Frontal_lobe_GM_L | 40.0 | 46.7 | 13.3 | 46.7 | 53.3 | 20.0 |
| Temporal_lobe_GM_L | 26.7 | 26.7 | 13.3 | 53.3 | 46.7 | 20.0 |
| Parietal_lobe_GM_L | 46.7 | 46.7 | 20.0 | 46.7 | 46.7 | 20.0 |
| Occipital_lobe_GM_L | 46.7 | 40.0 | 20.0 | 66.7 | 53.3 | 20.0 |
| Frontal_lobe_GM_R | 33.3 | 53.3 | 13.3 | 40.0 | 60.0 | 13.3 |
| Temporal_lobe_GM_R | 26.7 | 26.7 | 13.3 | 53.3 | 40.0 | 20.0 |
| Parietal_lobe_GM_R | 60.0 | 53.3 | 13.3 | 60.0 | 60.0 | 13.3 |
| Occipital_lobe_GM_R | 46.7 | 53.3 | 20.0 | 66.7 | 60.0 | 33.3 |
| Basal_ganglia_L | 40.0 | 40.0 | 13.3 | 40.0 | 53.3 | 20.0 |
| Basal_ganglia_R | 26.7 | 40.0 | 6.7 | 40.0 | 46.7 | 13.3 |
| Whole_brain_WM | 20.0 | 0.0 | 6.7 | 20.0 | 0.0 | 6.7 |
| Corp_Callosum | 13.3 | 13.3 | 13.3 | 20.0 | 13.3 | 20.0 |
| Brainstem | 6.7 | 0.0 | 6.7 | 20.0 | 6.7 | 20.0 |
| Cerebellum_l_wm | 6.7 | 6.7 | 0.0 | 13.3 | 13.3 | 6.7 |
| Cerebellum_r_wm | 6.7 | 0.0 | 0.0 | 13.3 | 6.7 | 13.3 |
| Frontal_lobe_WM_L | 20.0 | 13.3 | 6.7 | 26.7 | 6.7 | 6.7 |
| Temporal_lobe_WM_L | 20.0 | 13.3 | 13.3 | 20.0 | 13.3 | 20.0 |
| Parietal_lobe_WM_L | 20.0 | 6.7 | 6.7 | 20.0 | 6.7 | 6.7 |
| Occipital_lobe_WM_L | 26.7 | 13.3 | 20.0 | 33.3 | 20.0 | 20.0 |
| Frontal_lobe_WM_R | 26.7 | 0.0 | 6.7 | 33.3 | 0.0 | 6.7 |
| Temporal_lobe_WM_R | 40.0 | 20.0 | 13.3 | 40.0 | 20.0 | 13.3 |
| Parietal_lobe_WM_R | 26.7 | 6.7 | 6.7 | 26.7 | 6.7 | 6.7 |
| Occipital_lobe_WM_R | 40.0 | 33.3 | 20.0 | 33.3 | 33.3 | 26.7 |
|  |  |  |  |  |  |  |
| Total | 27.4 | 22.6 | 11.0 | 35.7 | 29.8 | 15.7 |

*GM = grey matter, WM = white matter, R = right, L = left

Supplementary Table 12: Assessment of the effect of acquisition time on the accuracy of the outcome parameter Ki as estimated from the 2T3k model. The percentage of subjects with %SE in the outcome parameter >25% are presented.

| **Acquisition time** | **40 min** | **50 min** | **60 min** |
| --- | --- | --- | --- |
|  |  |  |  |
| Whole_brain_GM | 0.0 | 0.0 | 0.0 |
| Thalamus_l | 6.7 | 0.0 | 0.0 |
| Thalamus_r | 6.7 | 6.7 | 0.0 |
| Cerebellum_l | 6.7 | 6.7 | 0.0 |
| Cerebellum_r | 6.7 | 0.0 | 0.0 |
| Frontal_lobe_GM_L | 0.0 | 0.0 | 0.0 |
| Temporal_lobe_GM_L | 6.7 | 0.0 | 0.0 |
| Parietal_lobe_GM_L | 0.0 | 0.0 | 0.0 |
| Occipital_lobe_GM_L | 0.0 | 0.0 | 0.0 |
| Frontal_lobe_GM_R | 0.0 | 0.0 | 0.0 |
| Temporal_lobe_GM_R | 0.0 | 0.0 | 0.0 |
| Parietal_lobe_GM_R | 0.0 | 0.0 | 0.0 |
| Occipital_lobe_GM_R | 0.0 | 0.0 | 0.0 |
| Basal_ganglia_L | 0.0 | 0.0 | 0.0 |
| Basal_ganglia_R | 0.0 | 0.0 | 0.0 |
| Whole_brain_WM | 0.0 | 0.0 | 0.0 |
| Corp_Callosum | 0.0 | 0.0 | 0.0 |
| Brainstem | 0.0 | 0.0 | 0.0 |
| Cerebellum_l_wm | 0.0 | 0.0 | 0.0 |
| Cerebellum_r_wm | 0.0 | 0.0 | 0.0 |
| Frontal_lobe_WM_L | 0.0 | 0.0 | 0.0 |
| Temporal_lobe_WM_L | 0.0 | 0.0 | 0.0 |
| Parietal_lobe_WM_L | 0.0 | 0.0 | 0.0 |
| Occipital_lobe_WM_L | 0.0 | 0.0 | 0.0 |
| Frontal_lobe_WM_R | 0.0 | 0.0 | 0.0 |
| Temporal_lobe_WM_R | 0.0 | 0.0 | 0.0 |
| Parietal_lobe_WM_R | 0.0 | 0.0 | 0.0 |
| Occipital_lobe_WM_R | 0.0 | 0.0 | 0.0 |
|  |  |  |  |
| Total | 1.2 | 0.5 | 0.0 |

*GM = grey matter, WM = white matter, R = right, L = left

Supplementary Table 13: The Ki estimated from the 2Tk3 model using different scan durations

| **Region** | **Ki** [mL min^-1^ cm^-3^] **[mean (SD)]** | | |
| --- | --- | --- | --- |
|  | **40 min** | **50 min** | **60 min** |
| Whole_brain_GM | 0.071 (0.022) | 0.066 (0.022) | 0.057 (0.016) |
| Thalamus_l | 0.109 (0.039) | 0.097 (0.037) | 0.079 (0.028) |
| Thalamus_r | 0.100 (0.032) | 0.086 (0.030) | 0.071 (0.026) |
| Cerebellum_l | 0.065 (0.023) | 0.060 (0.022) | 0.051 (0.017) |
| Cerebellum_r | 0.064 (0.022) | 0.059 (0.022) | 0.051 (0.017) |
| Frontal_lobe_GM_L | 0.074 (0.023) | 0.070 (0.023) | 0.061 (0.018) |
| Temporal_lobe_GM_L | 0.066 (0.021) | 0.062 (0.021) | 0.053 (0.017) |
| Parietal_lobe_GM_L | 0.077 (0.024) | 0.072 (0.024) | 0.063 (0.018) |
| Occipital_lobe_GM_L | 0.073 (0.025) | 0.070 (0.024) | 0.060 (0.016) |
| Frontal_lobe_GM_R | 0.076 (0.023) | 0.072 (0.023) | 0.063 (0.018) |
| Temporal_lobe_GM_R | 0.066 (0.022) | 0.061 (0.021) | 0.053 (0.016) |
| Parietal_lobe_GM_R | 0.074 (0.024) | 0.071 (0.024) | 0.062 (0.018) |
| Occipital_lobe_GM_R | 0.072 (0.024) | 0.069 (0.024) | 0.060 (0.016) |
| Basal_ganglia_L | 0.089 (0.027) | 0.084 (0.027) | 0.072 (0.021) |
| Basal_ganglia_R | 0.089 (0.025) | 0.082 (0.025) | 0.070 (0.021) |
| Whole_brain_WM | 0.131 (0.038) | 0.122 (0.036) | 0.103 (0.035) |
| Corp_Callosum | 0.116 (0.034) | 0.108 (0.032) | 0.093 (0.033) |
| Brainstem | 0.133 (0.042) | 0.119 (0.037) | 0.096 (0.035) |
| Cerebellum_l_wm | 0.147 (0.043) | 0.133 (0.039) | 0.111 (0.038) |
| Cerebellum_r_wm | 0.140 (0.042) | 0.129 (0.039) | 0.107 (0.036) |
| Frontal_lobe_WM_L | 0.132 (0.038) | 0.125 (0.038) | 0.106 (0.036) |
| Temporal_lobe_WM_L | 0.110 (0.033) | 0.104 (0.031) | 0.088 (0.029) |
| Parietal_lobe_WM_L | 0.128 (0.039) | 0.121 (0.037) | 0.104 (0.036) |
| Occipital_lobe_WM_L | 0.114 (0.032) | 0.107 (0.032) | 0.093 (0.027) |
| Frontal_lobe_WM_R | 0.133 (0.038) | 0.124 (0.038) | 0.107 (0.038) |
| Temporal_lobe_WM_R | 0.110 (0.032) | 0.103 (0.031) | 0.088 (0.029) |
| Parietal_lobe_WM_R | 0.125 (0.037) | 0.119 (0.037) | 0.103 (0.035) |
| Occipital_lobe_WM_R | 0.112 (0.031) | 0.107 (0.031) | 0.092 (0.026) |

*GM = grey matter, WM = white matter, R = right, L = left


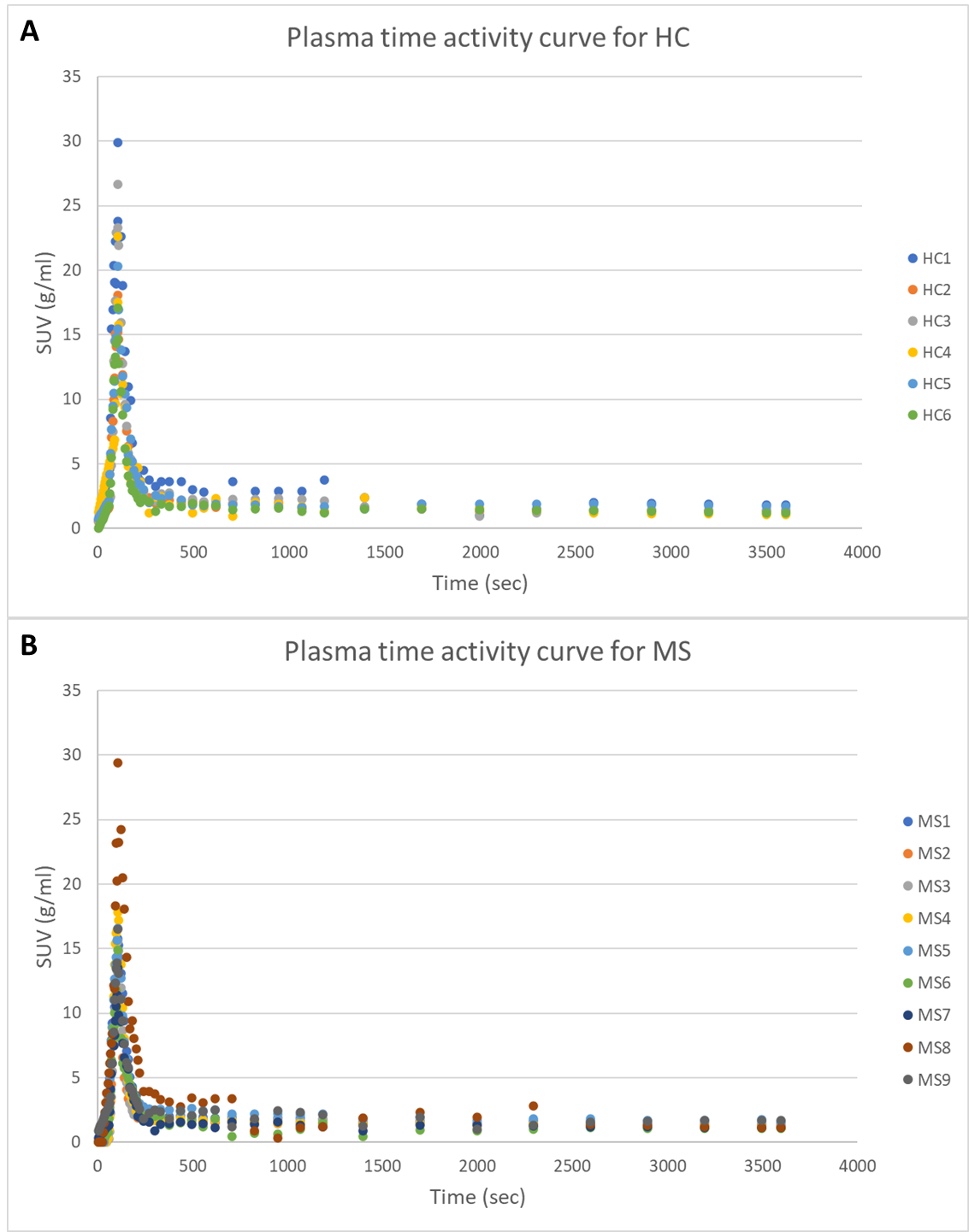


Supplementary Figure 1: Non metabolite corrected plasma time activity curves for (A) HC and (B) MS patients.


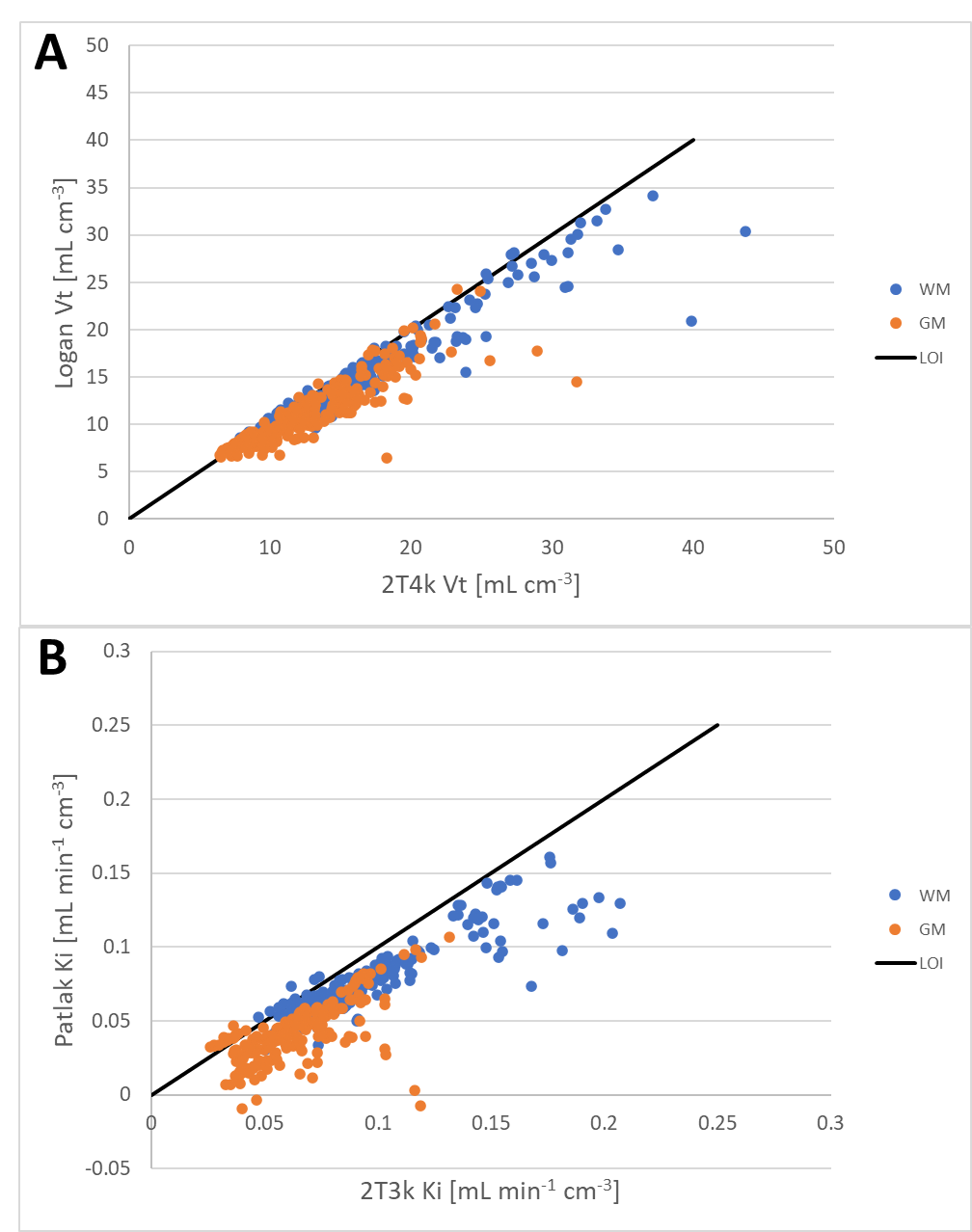


Supplementary Figure 2: Correlations between (A) Logan graphical analysis V_T_ and 2T4k V_T_ and (B) Patlak graphical analysis K_i_ and 2T3k K_i_. WM = white matter, GM = grey matter, LOI = line of identity
